# Clinical and Genetic Findings in Children with Neurofibromatosis Type 1, Legius Syndrome, and Other Related Neurocutaneous Disorders

**DOI:** 10.3390/genes10080580

**Published:** 2019-07-31

**Authors:** Teresa Giugliano, Claudia Santoro, Annalaura Torella, Francesca Del Vecchio Blanco, Anna Grandone, Maria Elena Onore, Mariarosa Anna Beatrice Melone, Giulia Straccia, Daniela Melis, Vincenzo Piccolo, Giuseppe Limongelli, Salvatore Buono, Silverio Perrotta, Vincenzo Nigro, Giulio Piluso

**Affiliations:** 1 Department of Precision Medicine, University of Campania “Luigi Vanvitelli”, Via L. De Crecchio 7, 80138 Napoli, Italy; 2 Departement of Women’s and Children’s Health and General and Specialized Surgery, University of Campania “Luigi Vanvitelli”, Via Luigi De Crecchio 2, 80138 Napoli, Italy; 3 Department of Medical Sciences and Advanced Surgery, University of Campania “Luigi Vanvitelli”, Piazza L. Miraglia 2, 80138 Napoli, Italy; 4 Department of Pediatrics, University of Naples “Federico II”, Via Pansini 5, 80131 Napoli, Italy; 5 Dermatology Unit, University of Campania “Luigi Vanvitelli”, Via Pansini 5, 80131 Napoli, Italy; 6 Department of Translational Medicine, University of Campania “Luigi Vanvitelli”, Via L. Bianchi c/o Ospedale Monaldi, 80131 Napoli, Italy; 7 Department of Neurosciences, "Santobono-Pausilipon" Pediatric Hospital, Via Fiore 6, 80129 Napoli, Italy; 8 Telethon Institute of Genetics and Medicine (TIGEM), Via Campi Flegrei 34, 80078 Pozzuoli, Italy

**Keywords:** neurofibromatosis type 1, Legius syndrome, RASopathies, next generation sequencing, multiplex ligation-dependent probe amplification, RNA analysis

## Abstract

Pigmentary manifestations can represent an early clinical sign in children affected by Neurofibromatosis type 1 (NF1), Legius syndrome, and other neurocutaneous disorders. The differential molecular diagnosis of these pathologies is a challenge that can now be met by combining next generation sequencing of target genes with concurrent second-level tests, such as multiplex ligation-dependent probe amplification and RNA analysis. We clinically and genetically investigated 281 patients, almost all pediatric cases, presenting with either NF1 (*n* = 150), only pigmentary features (café au lait macules with or without freckling; (*n* = 95), or clinical suspicion of other RASopathies or neurocutaneous disorders (*n* = 36). The causative variant was identified in 239 out of the 281 patients analyzed (85.1%), while 42 patients remained undiagnosed (14.9%). The *NF1* and *SPRED1* genes were mutated in 73.3% and 2.8% of cases, respectively. The remaining 8.9% carried mutations in different genes associated with other disorders. We achieved a molecular diagnosis in 69.5% of cases with only pigmentary manifestations, allowing a more appropriate clinical management of these patients. Our findings, together with the increasing availability and sharing of clinical and genetic data, will help to identify further novel genotype–phenotype associations that may have a positive impact on patient follow-up.

## 1. Introduction

Molecular diagnostic testing for Neurofibromatosis type 1 (NF1; MIM 162200) has improved considerably since identification of the first genotype–phenotype associations and an overlap disease, Legius syndrome (LS; MIM 611431) [1]. Both disorders belong to the RASopathies [2,3,4], a group of autosomal dominant and phenotypically overlapping disorders caused by mutations in genes encoding for components of the Ras/ mitogen activated protein kinase (MAPK) signaling pathway.

NF1 is a neurocutaneous condition characterized by multiple café au lait macules (CALMs), axillary and inguinal freckling, cutaneous neurofibromas, and iris Lisch nodules (LNs) [5,6]. Affected individuals show an increased susceptibility to developing benign tumors, such as plexiform neurofibroma, optic pathway glioma (OPG), and non-optic central nervous system glioma. Less common but potentially serious clinical manifestations are also reported [5,6,7].

With a birth incidence of approximately 1:3000, Neurofibromatosis type 1 is caused by dominantly inherited mutations in *NF1* (MIM 613113) [8], a complex gene [9] encoding for neurofibromin, a GTPase-activating protein that negatively regulates the Ras/MAPK signaling pathway [10]. Diagnosis of NF1 is still performed worldwide using clinical criteria formally codified in 1987 [6,11,12]. Nevertheless, clinical manifestations are variable and age related, with some distinguishing signs such as LNs and cutaneous neurofibromas appearing in late childhood or during puberty, further complicating clinical diagnosis of NF1 in young children, as well as in sporadic cases [6]. The differential diagnosis with other RASopathies is sometimes challenging due to the occurrence of signs of Noonan syndrome in NF1 patients (e.g., macrocephaly, Noonan-like facial features, short stature, and learning disabilities), as well as the presence of CALMs associated with some RASopathies (e.g., Noonan and LEOPARD syndromes) [13]. Milder NF1 phenotypes with pigmentary manifestations but without neurofibromas or OPG are associated with Met^992^ deletion and Arg^1809^ substitution in neurofibromin [14,15,16]. The 17q11.2 microdeletion fully encompassing *NF1* is known to be linked to a more severe phenotype with dysmorphic facial features, overgrowth or tall-for-age stature, significant delay in cognitive development, large hands and feet, hyper flexibility of joints, muscular hypotonia, and malignancies, such as the development of malignant peripheral nerve sheath tumor (MPNST) [17,18].

Initially described as an NF1-like phenotype, LS is caused by mutations in *SPRED1* (MIM 609291) [1], which encodes Spred1, a member of the Sprouty/Spred protein family [19] and, similarly to neurofibromin, a negative regulator of the Ras/MAPK signaling pathway [20,21]. Legius syndrome is characterized by CALMs and freckling without neurofibromas or other typical NF1 features, such as LNs, bony lesions, and OPGs [1,22,23]. 

Milder or incomplete NF1 phenotypes observed in young-aged patients, sometimes related to specific mildly pathogenic *NF1* variants, clinically overlap with LS or, in some cases, with other RASopathies and constitutional mismatch repair deficiency (CMMRD; MIM 276300) [24], necessitating recourse to molecular testing. In this diagnostic scenario, the increasing use of next generation sequencing (NGS), and particularly customized targeted gene panels, provides the opportunity to investigate these clinically overlapping conditions in a time- and cost-saving manner. The concurrent use of second-level tests, such as multiplex ligation-dependent probe amplification (MLPA) or RNA analysis by RT-PCR and Sanger sequencing, can be useful to highlight specific classes of variants or to precisely characterize the effect of each variant on the protein product. 

Here, we report our 10 year experience in molecular diagnosis of NF1 and LS, as well as other neurocutaneous conditions, in a large cohort of mostly pediatric patients. We also discuss how NGS and RNA analysis can improve the genetic characterization of patients, permitting differential diagnosis and guiding clinical follow-up. 

## 2. Materials and Methods

### 2.1. Patient Recruitment and Clinical Classification

A total of 281 subjects, including 164 males (58.4%) and 117 females (41.6%), most of which were children (mean age 14 ± 12 years at the pre-test medical examination), were recruited for this study mainly from the Neurofibromatosis Referral Center at the University of Campania “Luigi Vanvitelli” Department of Pediatrics. They were clinically evaluated according to the NIH diagnostic criteria and classified into six different groups. 

Typical pigmentary manifestations (CALMs with or without freckling) were considered as the main clinical sign in children and were combined with distinctive NF1 features (LNs, OPG, bone dysplasia, and neurofibromas), age at the pre-test medical examination, and presence of affected first-degree relatives. Of the 281 subjects involved in this study, 150 received a definite clinical diagnosis of NF1 due to the presence of at least one NF1 distinctive sign and were further molecularly characterized only on the parents’ request or in presence of a milder phenotype (*n* = 139; Group 1), or when an *NF1* microdeletion was suspected in the presence of a severe NF1 phenotype (*n* = 11; Group 2). 

An age-based categorization was established mainly to prioritize *NF1*/*SPRED1* mutation analysis, relying on the fact that some typical NF1 features, such as LNs and neurofibromas, may not be yet present in children aged <10 years. A further 44 patients with apparently pigmentary manifestations only, without affected first-degree relatives, and aged ≤ 9 years were prioritized for mutation analysis of *NF1* and, subsequently, *SPRED1* (Group 3), while 51 patients either with pigmentary manifestations only, without affected first-degree relatives, and aged ≥ 10 years (*n* = 31; Group 4), or with at least one affected first-degree relative (*n* = 20; Group 5) were prioritized for mutation analysis of *SPRED1* and subsequently *NF1*. Finally, 36 patients with clinical features suggestive of a RASopathy or other neurocutaneous disorders formed Group 6.

Samples were also collected from patients’ affected or unaffected relatives (*n* = 167) when necessary. 

Written informed consent for DNA analysis was obtained from all the subjects investigated or from their legal guardians at the pre-test medical examination, including explicit consent for future use of data for research purposes, according to the Declaration of Helsinki. Approval for the study was obtained from the Ethics Committee of the University of Campania “Luigi Vanvitelli” (#254-05/02/2019).

For each subject, blood samples were collected in PAXgene Blood RNA Tubes (Qiagen, Hilden, Germany) or Tempus Blood RNA Tubes (Life Technologies, Carlsbad, CA, USA) to prevent illegitimate splicing during subsequent RNA extraction and analysis [25,26]. Genomic DNA was also extracted using standard procedures.

### 2.2. Primer Design for NF1 and SPRED1 Mutation Screening

*NF1* pseudogenes occur on different human chromosomes [27]. To minimize the amplification of targets other than the expected templates, we used the Primer-BLAST tool (http://www.ncbi.nlm.nih.gov/tools/primer-blast/) for primer design.

For RNA analysis of the entire coding sequences of *NF1* and *SPRED1* (RefSeq: NM_000267.3 and NM_152594.3, respectively), we designed primer pairs that amplified partially overlapping fragments of 500–700 bp (Supplementary Materials Table S1). For both genes, genomic oligonucleotide pairs were also designed to amplify each exon and its intronic flanking regions (Supplementary Materials Table S2).

### 2.3. Mutation Screening by RT-PCR

For a large number of subjects investigated, *NF1* and *SPRED1* were analyzed at the cDNA level.

Total RNA was extracted using PAXgene Blood RNA Kit (Qiagen, Hilden, Germany) or Tempus Spin RNA Isolation Kit (Life Technologies, Carlsbad, CA, USA) according to the manufacturers’ specifications. RNAs were then retro-transcripted using SuperScript III RT (Invitrogen, Carlsbad, CA, USA) and random primers, according to the manufacturer’s instructions. Single-strand cDNAs were used in later experiments.

The RT-PCR was performed in a final volume of 20 μL containing 2 μL cDNA, 1X PCR Buffer II (Applied Biosystems, Foster City, CA, USA), 1 mM MgCl_2_, 1 mM dNTPs, 0.5 μM of each primer, and 0.5 U of AmpliTaq Gold DNA polymerase (Applied Biosystems). Cycling conditions consisted of a first step at 96 °C for 7 min followed by 30 cycles of 30 s at 96 °C, 1 min at 63 °C, and 3 min plus 3 s/cycle at 68 °C. 

The RT-PCR products were first analyzed by agarose gel electrophoresis to highlight possible unexpected products. For each sample, overlapping fragments covering the entire *NF1* or *SPRED1* coding sequence were subsequently analyzed by bidirectional sequencing. 

### 2.4. Targeted NGS-Based Mutational Screening

To extend mutation analysis to other genes involved in RASopathies, neurocutaneous disorders, and other genetically determined conditions with pigmentary manifestations in pediatric age, we designed a customized target NGS panel using HaloPlex technology (Agilent Technologies, Santa Clara, CA, USA). We selected 35 known disease-causing genes (Supplementary Table S3). The custom panel design also included genes identified as potential interactors of these 35 disease genes using two different bioinformatic tools, STRING and GeneMania [28,29]. Only genes matching both tools were added to the design. 

Enrichment of target sequences of all selected coding genes was performed using the HaloPlex Target Enrichment System for Illumina (Agilent Technologies, Santa Clara, CA, USA) according to the manufacturer’s instructions. For each sample, 200 ng of genomic DNA was digested with eight different restriction enzymes to create the fragment library and hybridized for 16 h to specific probes for Illumina sequencing. After capture of the biotinylated target DNA using streptavidin beads, nicks in the circularized fragments were closed by a ligase. Finally, the captured target DNA was eluted by NaOH and amplified by PCR. The amplified target molecules were purified using Agencourt AMPure XP beads (Beckman Coulter Genomics, Chaska, MN, USA). The enriched target DNA in each library sample was validated and quantified by microfluidic analysis using the Bioanalyzer High Sensitivity DNA Assay Kit and 2100 Bioanalyzer Expert Software (Agilent Technologies). Samples were run on a NextSeq500 System (Illumina, San Diego, CA, USA), generating 150 bp-long paired-end reads.

Generated sequences were analyzed using an in-house pipeline designed to automate the analysis workflow [30]. Average coverage for all the experiments was 70× and at least 20× for 98% of the target. Paired sequencing reads were aligned to the reference genome (UCSC, hg19 build) using a Burrows–Wheeler Aligner, and sorted with SAMtools and Picard (http://picard.sourceforge.net). Calling of single nucleotide variants (SNVs) and small insertions/deletions (Ins/Del) was performed with the Genome Analysis Toolkit (GATK) [31] with parameters adapted to HaloPlex-generated sequences. The called SNVs and Ins/Del variants were annotated using ANNOVAR [32], reporting variant position in RefSeq [33], amino acid change, presence in dbSNP v151 [34], frequency in the NHLBI Exome Variant Server (http://evs.gs.washington.edu/EVS), 1000 genomes [35], and Exome Aggregation Consortium (ExAC) browser (http://exac.broadinstitute.org) projects, multiple cross-species conservation [36], and prediction scores of damaging on protein activity [37]. 

### 2.5. Multiplex Ligation-Dependent Probe Amplification

To identify complete or partial deletions/duplications in *NF1*, *SPRED1, NF2, TSC1,* and *TSC2* genes, MLPA assays were performed using SALSA MLPA P081/P082 NF1 kit, SALSA MLPA P295 SPRED1 kit, SALSA MLPA P044 NF2 kit, SALSA MLPA P124 TSC1 kit, and SALSA MLPA P337 TSC2 kit, respectively (MRC-Holland, Amsterdam, The Netherlands), according to the manufacturer’s recommendations. When *NF1* microdeletions were detected, SALSA MLPA P122 NF1 kit (MRC-Holland) was also used to better define breakpoint boundaries. 

Briefly, denatured genomic DNA (100 ng) was added to the MLPA mix and the probes were allowed to anneal overnight before the subsequent ligation reaction was performed. Polymerase chain reaction (PCR) was carried out with 6-carboxyfluorescein (FAM)-labeled primers using 5 μL of the ligation reaction as the template. The PCR products were then separated on an ABI 3130xL automatic DNA sequencer (Life Technologies), including at least three normal DNA samples in each batch of the MLPA assays for the subsequent normalization of results. 

The MLPA data analysis was performed using the Coffalyser.Net package (MRC-Holland). Relative amounts of probe-amplified products were compared with reference samples to determine the copy number of target sequences. Values under a threshold of 0.7 and over a threshold of 1.3 for multiple adjacent probes indicate the presence of a deletion or duplication, respectively.

### 2.6. Real-Time PCR

To confirm copy-number mutations identified by MLPA, quantitative amplification of the specific genomic regions was performed on CFX96 Real-Time PCR Detection System (Bio-Rad Laboratories, Hercules, CA, USA) using iQ SYBR Green Supermix (Bio-Rad Laboratories) according to the manufacturer’s instructions. Uracil N-glycosylase (Amperase UNG, Life Technologies) was used to prevent PCR carry-over contamination. Each assay was performed in triplicate and the results were normalized and analyzed using CFX Manager software version 1.5 (Bio-Rad Laboratories, Hercules, CA, USA).

### 2.7. Validation of Variants by Sanger Sequencing

The PCR products were double-strand sequenced using BigDye Terminator sequencing chemistry (Life Technologies) and analyzed on an ABI 3130xL automatic DNA sequencer (Life Technologies, Carlsbad, CA, USA). Automatic variation calling was obtained by analyzing sequencing data (ABI file) using Mutation Surveyor software version 3.24 (SoftGenetics, State College, PA, USA), followed by careful inspection of the electropherograms to minimize variant loss.

## 3. Results

Over the last decade, approximately 600 patients suspected of being affected by NF1 or an NF1-like condition, or by RASopathies and other neurocutaneous disorders, were clinically evaluated at our Neurofibromatosis Referral Center in accordance with the NIH diagnostic criteria. For these patients, genetic testing was proposed whenever it may have been useful to confirm the clinical diagnosis. A total of 281 probands gave their informed consent and were included in this study, and molecular analysis was extended to their affected or unaffected relatives (*n* = 167) when necessary. To optimize the use of genetic testing in discriminating NF1 versus LS and other neurocutaneous disorders in childhood, patients were classified into six groups (see Materials and Methods) and prioritized according to their clinical features.

### 3.1. Molecular Diagnosis

Results of molecular diagnosis for each patient group are summarized in Table 1. The causative variant was detected in 239 out of 281 patients analyzed (85.1%), with only 42 undiagnosed patients (14.9%). Both *NF1* and *SPRED1* were mutated in 73.3% and 2.8% of cases, respectively. The remaining 8.9% presented causative variants in different genes.

In subjects with a clinical diagnosis of NF1 (Groups 1, 2), the mutation detection rate was 98% (147/150), similarly to previously reported findings [38]. When pigmentary manifestations were the only early clinical sign (Groups 3–5), the mutation detection rate fell to 69.5% (66/95), with *SPRED1* accounting for around 8.4% (8/95) of identified causative variants. Interestingly, the lowest detection rate (64.5%) was obtained for subjects presenting only CALMs, aged ≥ 10 years at the pre-test medical examination, and without affected first-degree relatives (Group 4). In contrast, subjects presenting only CALMs and with at least one affected first-degree relative (Group 5) achieved a higher mutation detection rate (85%), with *NF1* and *SPRED1* accounting for 55% and 30%, respectively.

In subjects with a clinical suspicion of a RASopathy or neurocutaneous disorder, the mutation detection rate was 72.2% (26/36), with variants distributed in ten different genes, mainly *PTPN11* (22.2%), and with only one causative variant in *NF1* (2.7%). 

### 3.2. NF1 Mutation Screening

By combining NGS, direct sequencing of RT-PCR products, and MLPA analysis, we identified 169 different causative variants along *NF1* (Table A1). As expected, 33.1% of these (56/169) were novel variants. Single-nucleotide substitutions and single or very short deletion/insertion of bases accounted for 67.4% (114/169) and 30.2% (51/169) of the identified causative variants, respectively. Wider deletions or duplications at *NF1* locus made up the remaining 2.4%. Excluding this last class of mutations, variants were distributed in almost all *NF1* exons (Supplementary Materials Figure S1) and only 21 were recurrent variants, being present in at least two unrelated patients.

Base substitutions resulted in 31 nonsense variants (27.2%), three of which were novel, 34 missense variants (29.8%), eight of which were not previously reported, and 42 variants differently affecting splicing (36.8%), mainly resulting in a frameshift of *NF1* coding sequence.

Novel missense variants were further investigated, considering segregation in familial cases or their de novo occurrence. In support of their pathogenic effect, these amino acid changes were predicted to be deleterious by common in silico prediction programs (SIFT, Polyphen-2, and Mutation Taster; Supplementary Materials Table S4) [39,40,41], and were not annotated in the gnomAD and ExAC browsers [42].

Variants affecting mRNA splicing are common in *NF1* [25,38]. In our study, they represent 25.4% (43/169) of all identified causative variants, with 34 variants differently perturbing canonical splice acceptor or donor sites, five variants within exons creating de novo splice sites and resulting in the loss of a part of the exon, and four deep intronic mutations activating cryptic splice sites (Supplementary Materials Table S5). This last type would likely be underestimated without RNA analysis. In our cohort of NF1 patients, this class of mutation accounted for 2.4% of all identified variants (4/169), three of which were not previously reported.

Complete *NF1* microdeletion and other rearrangements partially involving *NF1* were detected by MLPA analysis. In line with other reports [43,44], *NF1* microdeletions at 17q11.2 were present in 4.9% (10/205) of patients with an identified causative variant in *NF1*, while two intragenic deletions of exons 15(11)–36(27b) and exons 28(22)–29(23) and a duplication of exons 37(28)–51(42) in *NF1* were identified in three further patients.

### 3.3. SPRED1 Mutation Screening

We identified eight different causative variants in *SPRED1* (Table A2), three of which were novel. Single nucleotide substitutions resulted in four already reported nonsense variants and one missense variant. We also identified a novel 5 bp deletion in a large family with 10 affected individuals and a novel one-base duplication in another family. The MLPA analysis characterized a sporadic case with an intragenic deletion of the last two exons and the 3’UTR of *SPRED1*.

### 3.4. Phenotype-Genotype Overview

Table A3 summarizes the clinical features of 245 probands suspected of being affected by NF1 or an NF1-like condition evaluated at the pre-test medical examination (T0) and after genetic testing (T1). Based on the NIH diagnostic criteria, a clinical diagnosis of NF1 was achieved in 150 patients (Groups 1 and 2), 99.3% of which presented CALMs either with (78.7%) or without (18%) freckling associated with LNs (55.4%), OPG (14%), bone dysplasia (2%), cutaneous or plexiform neurofibromas (62.7%), or an affected first-degree relative (41.3%). One case (Family ID 108) did not quite meet the NIH diagnostic criteria but was nevertheless included in Group 1 due to the presence of neurofibromas at age 5 months, and minor clinical features such as macrocephaly, nevus anemicus, psychomotor delay, and thorax abnormalities. About 98% of these clinically diagnosed patients presented a causative *NF1* variant that resulted in truncated or absent neurofibromin (75.5%), or in in-frame deletions (10.9%) or single substitutions (13.6%) of amino acids.

The remaining 95 patients (Groups 3–5) only presented CALMs (100%), with (29.5%) or without (70.5%) freckling and were negative for the other NIH diagnostic criteria at the pre-test medical examination. Among these, 75 patients were sporadic cases aged ≤ 9 years (*n* = 44; Group 3) or ≥10 years (*n* = 31; Group 4), while the remaining 20 patients were familial cases (Group 5). In Group 3, a causative variant in *NF1* was identified in 63.6% of patients, resulting in truncated or absent neurofibromin (67.9%), or in in-frame deletions (10.7%) or single substitutions (21.4%) of amino acids. In Group 4, which presented the lowest mutation detection rate (61.3%), *NF1* was still the most commonly involved gene with an increased percentage of variants causing in-frame deletions or single substitutions of amino acids (61.1%) compared to those resulting in truncated or absent neurofibromin (38.9%). Only one causative variant was detected in *SPRED1* (Family ID 157). In Groups 3 and 4, typical NF1 clinical features subsequently appeared in only 11 patients with a causative variant in *NF1* identified by genetic testing (T1). For Groups 3 and 4, 48% of patients (36/75) presented only CALMs without any other typical NF1 feature, even after genetic testing (T1), thus not falling within the NIH diagnostic criteria. Interestingly, a causative variant in *NF1* was identified in 30.6% of cases (11/36). In only one case (Family ID 224) was a causative variant in *SPRED1* detected. Finally, *NF1* and *SPRED1* were similarly mutated in patients from Group 5. Variants in *NF1* (55%) gave rise to truncated or absent neurofibromin in only two patients, while *SPRED1* variants (30%) mainly caused haploinsufficiency. Again, in Group 5, no further typical NF1 clinical features subsequently appeared in patients with an *NF1* variant identified by genetic testing (T1).

Neurofibromatosis bright objects were the most frequently observed of all minor clinical features (Table A3), presenting in 20.8% of cases. Learning disabilities and/or speech problems were found in 18.8% of patients, while thorax abnormalities, macrocephaly, leg length discrepancy, and scoliosis in 16.7%. Noonan-like facial features (7.8%), intellectual disability (6.9%), and behavior problems (5.7%) were also observed. Less common but potentially serious malignancies, including malignant peripheral nerve sheath tumor (MPNST), leukemia, and rhabdomyosarcoma, accounted for 2.9% of cases, while vascular alterations such as Moyamoya syndrome and pulmonary stenosis were observed in 2% and 1.6% of cases, respectively.

### 3.5. Mutation Screening in Non-NF1 or NF1-Like Conditions and Unsolved Cases

By combining NGS and MLPA analysis, we also investigated 36 patients with clinical features suggestive of a RASopathy or neurocutaneous disorder (Group 6; Table A4). Among these, 14 patients with RASopathy features presented causative variants in *PTPN11* (8/14), *SOS1* (2/14), *PPP1CB* (1/14), and *NF1* (1/14), 14 patients diagnosed with tuberous sclerosis complex (TSC) showed variants in *TSC1* (3/14) and *TSC2* (5/14), five patients with Neurofibromatosis type 2 or Schwannomatosis presented causative variants in *NF2* (3/5) and *LZTR1* (1/5), while a variant in *PTEN* and *KIT* was identified in two other cases with a clinical diagnosis of Cowden syndrome and Piebaldism. Six of the identified causative variants were not previously reported (Table A4 and Supplementary Materials Table S4). Biallelic germline variants in mismatch repair (MMR) genes are known to be responsible for CMMRD. Although this condition is associated with a broad spectrum of early-onset tumors often associated with NF1 features, especially CALMs [24,45], no causative variants in MMR genes were found in our cohort. 

In 42 unsolved cases, we also investigated for variants in candidate genes, considering different models of inheritance. For one patient only, we identified a rare missense heterozygous variant in *MAPK3* (NM_001109891.1:c.601C>A; p.Leu201Met) not present in our internal database or in any public databases. Although *MAPK3* encodes for a member of the MAP kinase family [46], any pathogenic role for the observed variant is currently only speculative.

## 4. Discussion

In recent years, NGS has greatly improved the molecular diagnosis of inherited diseases, particularly in the case of genetically heterogeneous and clinically overlapping conditions. Our experience further supports the diagnostic value of NGS and shows how a targeted NGS-based entry-level test [47,48,49] combined with RNA and MLPA analysis for a complete molecular characterization [50,51] achieves a high mutation detection rate and is extremely useful in addressing differential diagnosis of NF1 and overlap diseases. In fact, we obtained a molecular diagnosis in about 85% of cases investigated.

For patients with a clinical diagnosis of NF1 (Groups 1, 2), 98% carried an *NF1* causative variant, resulting in truncated or absent neurofibromin in 75.5% of cases. All subjects presented CALMs, with freckling in 86% of cases, as well as the most common typical NF1 features including neurofibromas (64.7%), LNs (59.4%), and OPG (16%). Recently, causative variants in the cysteine/serine-rich domain (CSRD; residues 543–909) were positively associated with OPG [52]. Among the 24 NF1 patients presenting OPG investigated, seven showed an *NF1* variant within the CSRD domain. Intellectual and/or learning disability or speech problems were present in about 26.7% of cases, while 8.7% showed Noonan-like facial features [53]. Malignancies and vascular alterations, such as Moyamoya syndrome, were observed in 4% and 4.7% of cases, respectively [54]. A large NF1 family with co-occurrence of Moyamoya syndrome in two first cousins (Family ID 16) was recently further investigated by whole exome sequencing, which identified *MRVI1* as a susceptibility gene for Moyamoya syndrome in NF1 [55]. Of 11 patients with a more severe NF1 phenotype (Group 2) suggestive of *NF1* microdeletion [17,18,56], a 17q11.2 microdeletion was in fact detected in six cases. Among these, two patients later died from MPNST, frequently seen in NF1 microdeletion patients [18,56]. Truncating variants were present in four other cases with severe NF1 phenotype, and removed large part of the protein sequence with its functional domains. The remaining patient (Family ID 119) showed an in-frame deletion (p.Tyr1614_Tyr1618del) falling in the Sec14-like domain of neurofibromin. This patient showed moderate intellectual disability with learning difficulties and speech problems, macrocephaly, dysmorphic facial features, tall stature, and skeletal anomalies (leg length discrepancy, dystrophic scoliosis, and vertebral scalloping), a small number of subcutaneous neurofibromas, and medullary unidentified bright objects [57]. Interestingly, the Sec14-like domain of neurofibromin interacts with valosin-containing protein (VCP), regulating dendritic spine density [58]. Dominantly inherited *VCP* mutations cause inclusion body myopathy with Paget disease of bone and frontotemporal dementia [59].

Molecular investigation was critical in achieving a clinical diagnosis for patients with only pigmentary features (CALMs with or without freckling; Groups 3–5); for these patients we were able to detect the causative variant in 69.5% of cases. This result highlights the clinical utility of genetic testing, particularly in pediatric age, even in those cases which do not fall within the NIH diagnostic criteria, driving the patient’s clinical early follow-up and management. In sporadic cases (*n* = 75; Groups 3, 4), *NF1* was mutated in 62.7% of patients, with only two (Family ID 157 and 224) presenting a variant in *SPRED1* causing haploinsufficiency. Among the *NF1* variants, 55.3% resulted in haploinsufficiency, while the rest were in-frame deletions or single substitutions of amino acids. Subsequent to genetic testing, additional typical NF1 features (LNs and/or neurofibromas) appeared in 11 NF1 patients, 10 of which had a truncating variant in *NF1*. The remaining NF1 patients presented a mild phenotype, with only CALMs either with or without freckling, in some cases complicated by speech and learning problems, short stature, and macrocephaly. Of these, three cases carried the Arg^1809^ substitution in neurofibromin [15,16]. 

The causative variant was detected in 85% of patients with pigmentary manifestations also present in at least one affected first-degree relative (*n* = 20; Group 5), with *NF1* and *SPRED1* similarly involved. Missense variants were the most common type of variants found in *NF1*, with the Arg^1809^ substitution accounting for 45.5% [15,16]. In Group 5, we also molecularly diagnosed the highest percentage of LS (30%), which appears to be primarily associated with inherited mutant alleles, unlike NF1, in which de novo variants frequently occur.

Noonan-like features can be observed in NF1 patients. A neurofibromatosis-Noonan Syndrome (MIM 601321) was reported [60,61] and linked to variants in *NF 1* [53], but also to the co-occurrence of independent variants in *NF1* and *PTPN11* in the same patient [62,63]. The NGS analysis excluded additive variants in Noonan syndrome-causing genes in our molecularly diagnosed NF1 patients with combined NF1 and Noonan-like features.

We also extended our investigation to 36 patients with clinical features suggestive of other RASopathies or neurocutaneous disorders (Group 6), identifying a causative variant in line with clinical suspicion in 25 cases (Table A4). In one child (Family ID 284) referred by endocrinologists due to clinical suspicion of Noonan syndrome, we did not find any causative variants in Noonan syndrome-causing genes but, unexpectedly, an unreported and maternally inherited missense variant in *NF1* (p.Glu1198Lys). At genetic counseling, the patient presented CALMs, Noonan-like facial features and habitus (short stature, relative macrocephaly, hypertelorism, thoracic asymmetry, and sternum carinatum). Her mother also presented a very small number of CALMs, mild Noonan-like facial features, soft hands and feet, and short stature. Further investigation of this mild phenotype in other patients with the same *NF1* variant may help characterize a possible genotype-phenotype association. This case is illustrative of the diagnostic overlap between these two conditions, as well as in the recently reported case [64] of a child (Family ID 187) carrying an *SOS1* variant inherited from his mother, who initially received a diagnosis of NF1 due to the spinal nerve enlargement resembling neurofibromas. 

The combined or alternative use of NGS and RNA analysis was able to precisely characterize the functional effect of each causative variant identified in *NF1* and *SPRED1*, in some cases overcoming the limits of each approach when performed individually. Specifically, some missense or nonsense variants in *NF1* caused exon skipping or generated cryptic splice sites. Moreover, similarly exon-skipped *NF1* transcripts were caused by different genomic variants proximal to the same splice site. For some of these skipped exons, including 15(11), 37(27a), and 46(37), phenotype variability was observed in affected patients. Deep intronic mutations activating cryptic splice sites were only detectable by RNA analysis and accounted for 2.4% of all the causative variants we identified. Conversely, three variants in the first exon of *NF1* causing RNA decay were only detectable by NGS analysis. Considering recently reported (and potential novel) genotype-phenotype correlations [15,16,18,52,65,66,67,68], evaluating the functional effect of genomic variants can have a positive impact on patients’ clinical follow-up. 

Of the unsolved cases, only four patients had a definite clinical diagnosis of NF1 due to the presence of typical pigmentary manifestations combined with at least one additional distinctive NF1 clinical feature. One of these (Family ID 114) was subsequently diagnosed as having a mosaic form of NF1 because of the peculiar distribution of CALMs restricted to the right side of the trunk and LNs only in the right eye. As suggested by the lowest detection rate, isolated CALMs (Groups 3–5) are associated with a lower possibility of obtaining a molecular diagnosis. This might be due to mosaicism or to the existence of other genetic causes of isolated pigmentary manifestations yet to be discovered. In Group 6, six unsolved cases had a clinical diagnosis of TSC. However, about 15%–20% of TSC patients may have unidentifiable mutations or a mosaicism [69,70]. Finally, one patient (Family ID 219) with a clinical diagnosis of Schwannomatosis and negative for mutations in *NF2*, *LZTR1,* and *SMARCB1* is under investigation for a somatic mosaicism in *NF2*.

## 5. Conclusions

Our findings highlight the clinical and diagnostic challenges of a pediatric referral center for neurocutaneous disorders, demonstrating how a combined NGS-based approach can assist clinicians in the diagnosis of NF1 as well as other neurocutaneous disorders and overlapping conditions. We categorized patients based on clinical signs and considered an NF1 diagnosis certain only when other distinctive signs besides CALMs and freckling were present, achieving a very high detection rate and providing a precise characterization of identified causative variants. Our results also highlight how it can still make sense to prioritize patients for *NF1* mutation analysis when presenting only CALMs, typical of NF1 in term of number and diameter, and independently from the age at clinical observation. Our categorization suggests that older patients showing only CALMs tend to remain without a definite molecular diagnosis. The RNA analysis facilitates in interpreting the functional effect of genomic variants and can drive the identification of new genotype-phenotype correlations, potentially impacting on the clinical management of NF1 pediatric patients. Through the sharing of clinical and molecular data among members of the scientific community, we are confident it will be possible to identify novel genotype–phenotype correlations and ultimately improve patient outcomes.

## Figures and Tables

**Table 1:** Results of the molecular diagnosis by patient group.

Group	Criteria for Molecular Testing	Number of Selected Patients	Number of Mutated Patients	Mutation Detection Rate (%)	Number of Patients without Molecular Diagnosis (%)
1	Clinical diagnosis of NF1 (test requested by parents or milder phenotype)	139	136 (*NF1* = 136; *SPRED1* = 0; OTHER = 0)	97.8% (*NF1* = 97.8%; *SPRED1* = 0.0%; OTHER = 0.0%)	3 (2.2%)
2	Severe NF1 phenotype with suspicion of 17q11.2 microdeletion	11	11 (*NF1* = 11; *SPRED1* = 0; OTHER = 0)	100.0% (*NF1* = 100.0%; *SPRED1* = 0.0%; OTHER = 0.0%)	0 (0.0%)
3	Isolated CALMs in patients without affected first-degree relatives and age ≤ 9 y	44	29 (*NF1* = 28; *SPRED1* = 1; OTHER = 0)	65.9% (*NF1* = 63.6%; *SPRED1* = 2.3%; OTHER = 0.0%)	15 (34.1%)
4	Isolated CALMs in patients without affected first-degree relatives and age ≥ 10 y	31	20 (*NF1* = 19; *SPRED1* = 1; OTHER = 0)	64.5% (*NF1* = 61.3%; *SPRED1* = 3.2%; OTHER = 0.0%)	11 (35.5%)
5	Isolated CALMs in patients and at least one affected first-degree relative	20	17 (*NF1* = 11; *SPRED1* = 6; OTHER = 0)	85.0% (*NF1* = 55.0%; *SPRED1* = 30.0%; OTHER = 0.0%)	3 (15.0%)
6	Other RASopathies or neurocutaneous disorders	36	26 (*NF1* = 1; *SPRED1* = 0; OTHER = 25)	72.2% (*NF1* = 2.7%; *SPRED1* = 0.0%; OTHER = 69.4%)	10 (27.8%)
	Total	281	239 (*NF1* = 206; *SPRED1* = 8; OTHER = 25)	85.0% (*NF1* = 73.3%; *SPRED1* = 2.8%; OTHER = 8.9%)	42 (15.0%)

Notes: NF1 = neurofibromatosis type 1; CALMs = café au lait macules; OTHER = other disease genes investigated (the identified causative variants are reported in Table A4).

## Supplementary Materials

The following are available online at https://www.mdpi.com/2073-4425/10/8/580/s1, Figure S1: Distribution of identified variants in exons of *NF1*, Table S1: List of primer pairs designed to amplify overlapping fragments for RNA analysis of the entire coding sequences of *NF1* and *SPRED1*, Table S2: List of genomic primer pairs designed to amplify exons and intronic flanking regions of *NF1* and *SPRED1*, Table S3: List of genes included in the customized target NGS panel, Table S4: In silico prediction of deleterious effects and segregation analysis for unreported missense variants, Table S5: In silico prediction of splice score for deep intronic mutations in *NF1* (www.fruitfly.org/seq_tools/splice.html).

## Appendix A

**Table A1:** Unique variants identified in *NF1* (RefSeq NM_000267.3).

Family ID ^1^	Group	Exon	Type ^2^	Genomic	cDNA	Effect	Protein	ClinVar/HGMD/LOVD ID^3^
216 (3)	5	01(01)	SNV	3G>A	RNA decay	RNA decay	?	LOVD: NF1_001130
220 (1)	1	01(01)	SNV	59A>C (splicing)	RNA decay	RNA decay	?	New
212 (1)	1	01(01)	SNV	60G>C (splicing)	RNA decay	RNA decay	?	New
35 (2)	1	02(02)	SNV	128T>C	128T>C	Missense	Leu43Pro	LOVD: NF1_000049
67 (1)	1	03(03)	SNV	277T>C	277T>C	Missense	Cys93Arg	LOVD: NF1_002264
294 (1)	3	03(03)	SNV	288+1delG	288del	Splicing	Glu97Asnfs*6	LOVD: NF1_001615
81 (1)	3	03(03)	SNV	288+1G>A (Splicing)	205_288del	Splicing	Arg69_Gly96del	LOVD: NF1_001484
262 (1)	5	03(03)	SNV	288+4A>G	205_288del	Splicing	Arg69_Gly96del	LOVD: NF1_000270
93 (3)	1	03(03)	DIM	289-2956C>T (cryptic splice site)	288_289insAGTCTCACTCTGCGGCACAGGCTGAAGTGCAGTGGCACCCTCTCGGCTCATTGCAACCTCCACTTCCCGGGTTCAAGCTATTCTCATGCCTCAGCCTCCCAAGTAGCTGGGATTACAG	Intronic cryptic splice site	Gln97Valfs*8	New
72 (1)	1	04(04a)	DEL	363del	363del	Frame-shift	His122Thrfs*43	New
31 (1)	1	05(04b)	DEL	499_502del	499_502del	Frame-shift	Cys167Glnfs*10	LOVD: NF1_000605
251 (1)	1							
2 (1)	1	05(04b)	SNV	574C>T	574C>T	Nonsense	Arg192*	LOVD: NF1_000702
68 (1)	1							
121 (1)	3	07(05)	SNV	667T>A	667T>A	Missense	Trp223Arg	LOVD: NF1_000800
128 (1)	3	08(06)	SNV	818T>C	818T>C	Missense	Leu273Pro	New
221 (1)	1	09(07)	DEL	1019_1020del	1019_1020del	Frame-shift	Ser340Cysfs*12	LOVD: NF1_000005
172 (1)	1	10(08)	DEL	1110del	1110del	Frame-shift	Ala371Glnfs*5	New
49 (1)	3	10(08)	DEL	1123del	1123del	Frame-shift	Leu375*	New
296 (1)	4	10(08)	SNV	1144T>C	1144T>C	Missense	Ser382Pro	New
46 (1)	1	10(08)	SNV	1185+1G>A (splicing)	1063_1185del	Splicing	Asn355_Lys395del	LOVD: NF1_000019
65 (1)	1							
218 (1)	1	10(08)	SNV	1185G>T (splicing)	1063_1185del	Splicing	Asn355_Lys395del	HGMD: CS147216
125 (1)	3	11(09)	SNV	1186-3T>G (splicing)	1185_1186dup	Splicing	Ile396Glufs*17	New
292 (1)	1	11(09)	SNV	1198C>T	1198C>T	Nonsense	Gln400*	LOVD: NF1_000024
110 (1)	3	11(09)	INS	1243dup	1243dup	Frame-shift	His415Profs*14	ClinVar: 426651
23 (2)	1	11(09)	SNV	1246C>T	1246C>T	Nonsense	Arg416*	LOVD: NF1_000034
152 (1)	1							
45 (1)	3	11(09)	DIM	1260+1604A>G (cryptic splice site)	1260_1261insCTGACTACATAGAGCACTTTCAAGCATGGACTTGGCACTGCT	Intronic cryptic splice site	Ser421Leufs*4	LOVD: NF1_000035
165 (1)	1	11(09)	SNV	1260+1G>A	1260_1261insATAAGTCCAAAAG	Splicing	Ser421Ilefs*12	LOVD: NF1_000036
289 (1)	1	12(10a)	SNV	1261-2A>C (cryptic splice site)	1261_1284del	Splicing	Ser421_Lys428del	LOVD: NF1_000045
82 (1)	1	12(10a)	SNV	1318C>T	1318C>T	Nonsense	Arg440*	LOVD: NF1_000052
153 (1)	1							
54 (1)	4	12(10a)	DEL	1329del	1329del	Frame-shift	Phe443Leufs*30	LOVD: NF1_001174
52 (1)	1	12(10a)	INS	1378dup	1378dup	Frame-shift	Ile460Asnfs*10	New
21 (1)	1	12(10a)	SNV	1381C>T	1381C>T	Nonsense	Arg461*	LOVD: NF1_000056
57 (1)	3							
144 (3)	1							
160 (1)	1	12(10a)	DIM	1393-1554C>G (cryptic splice site)	1392_1393insTGAAGATTTGTTTACACCAGCATCACTACAAACAATAACGCATTGTGCTTGGACATCACGATGGCTATGATA	Intronic cryptic splice site	Ser465*	New
17 (3)	1	13(10b)	DEL	1393-2del	1393_1527del	Splicing	Ser465_Cys509del	LOVD: NF1_000061
116 (1)	1	13(10b)	INS	1399dup	1399dup	Frame-shift	Thr467Asnfs*3	LOVD: NF1_002283
288 (1)	1	13(10b)	DEL	1423del	1423del	Frame-shift	Lys476Asnfs*22	New
13 (1)	4	13(10b)	SNV	1466A>G (cryptic splice site)	1466_1527del	Splicing	Tyr489*	ClinVar: 354
222 (1)	1							
159 (1)	1	13(10b)	INS	1470_1471insATACG	1470_1471insATACG	Frame-shift	Tyr491Ilefs*9	New
132 (2)	1	13(10b)	SNV	1487T>G	1487T>G	Missense	Met496Arg	New
174 (1)	1	13(10b)	INDEL	1499_1501delinsAAA	1499_1501delinsAAA	DelIns	Ile500_His501delinsLysAsn	New
107 (1)	2	13(10b)	DEL	1500del	1500del	Frame-shift	His501Metfs*25	New
138 (1)	3	13(10b)	DIM	1527+1165T>A (cryptic splice site)	1527_1528insGATGACATGTTTAACCTTTGTTGAGCTTCTTCAGTCCCTGGAGAGCAGCATCAAGCAAG	Intronic cryptic splice site	Asn510Aspfs*7	New
76 (1)	1	14(10c)	DEL	1541_1542del	1541_1542del	Frame-shift	Gln514Argfs*43	LOVD: NF1_000074
44 (3)	1	14(10c)	SNV	1595T>G	1595T>G	Missense	Leu532Arg	LOVD: NF1_002498
5 (1)	4	15(11)	SNV	1642-1G>A (splicing)	1642_1721del	Splicing	Ala548Leufs*13	LOVD: NF1_000084
147 (3)	5	15(11)	SNV	1642G>T	1642_1721del	Splicing	Ala548Leufs*13	New
59 (1)	4	15(11)	SNV	1658A>G	1658A>G	Missense	His553Arg	LOVD: NF1_000091
186 (1)	1	15(11)	SNV	1721+3A>G (Splicing)	1642_1721del	Splicing	Ala548Leufs*13	LOVD: NF1_000101
85 (1)	1	15(11)	SNV	1721G>C (Splicing)	1642_1721del	Splicing	Ala548Leufs*13	New
150 (1)	1	15-36	DEL	1642-?_4772+?del (intragenic deletion ex. 15-36)	1642_4772del	Intragenic deletion	Ala548Valfs*9	New
15 (1)	1	16(12a)	SNV	1748A>G (cryptic splice site)	1722_1748del	Splicing	Ser574_Lys583delinsArg	LOVD: NF1_000110
102 (2)	1	17(12b)	SNV	1846C>T	1846C>T	Nonsense	Gln616*	LOVD: NF1_000125
133 (2)	1	17(12b)	DEL	1863del	1863del	Frame-shift	Cys622Valfs*9	LOVD: NF1_001427
168 (1)	2							
149 (1)	2							
214 (1)	1	17(12b)	SNV	1942G>T	1942G>T	Nonsense	Glu648*	LOVD: NF1_002848
55 (2)	1	17(12b)	INS	1995dup	1995dup	Frame-shift	Ser666Leufs*4	New
217 (1)	1	18(13)	SNV	2002-10T>A (cryptic splice site)	2001_2002insACTCTCAG	Splicing	Asp668Thrfs*23	New
8 (1)	1	18(13)	SNV	2002-1G>A (cryptic splice site)	2002_2011del	Splicing	Asp668Glnfs*17	LOVD: NF1_000143
192 (1)	1	18(13)	INDEL	2027_2028delinsA	2027_2028delinsA	DelIns	Thr676Asnfs*12	New
29 (2)	1	18(13)	INS	2033dup	2033dup	Frame-shift	Ile679Aspfs*21	LOVD: NF1_000148
63 (1)	1	18(13)	SNV	2041C>T	2041C>T	Nonsense	Arg681*	LOVD: NF1_000153
39 (2)	1	18(13)	INS	2167dup	2167dup	Frame-shift	Val723Glyfs*3	New
27 (2)	1	18(13)	SNV	2251G>C (splicing)	2002_2251del	Splicing	Asp668Glufs*9	ClinVar: 584927
209 (1)	1	19(14)	SNV	2266C>T	2252_2325del	Splicing	Arg752Leufs*17	LOVD: NF1_000174
75 (1)	1	19(14)	SNV	2288T>C	2288T>C	Missense	Leu763Pro	LOVD: NF1_000177
7 (2)	1	19(14)	INS	2307dup	2307dup	Frame-shift	Thr770Hisfs*6	New
53 (1)	3	20(15)	SNV	2326G>A	2326_2409del	Splicing	Trp777_Ala804del	New
126 (1)	1							
184 (2)	1	20(15)	SNV	2339C>A	2339C>A	Missense	Thr780Lys	LOVD: NF1_000190
234 (2)	1	20(15)	SNV	2339C>G	2339C>G	Missense	Thr780Arg	LOVD: NF1_001397
6 (3)	1	20(15)	SNV	2351G>C	2351G>C	Missense	Trp784Ser	LOVD: NF1_000196
33 (2)	1	20(15)	SNV	2352G>C	2352G>C	Missense	Trp784Cys	LOVD: NF1_001853
106 (1)	4	21(16)	SNV	2540T>G	2540T>G	Missense	Leu847Arg	LOVD: NF1_000229
188 (1)	1							
300 (1)	1	21(16)	SNV	2557C>T	2557C>T	Nonsense	Gln853*	LOVD: NF1_001222
241 (1)	1							
38 (1)	1	21(16)	SNV	2693T>C	2693T>C	Missense	Leu898Pro	LOVD: NF1_000241
48 (1)	1	21(16)	SNV	2850+1G>A (cryptic plice site)	2707_2850del	Splicing	Cys904_Val951del	LOVD: NF1_000259
18 (1)	1	22(17)	SNV	2851G>T (splicing)	2851_2990del	Splicing	Leu952Cysfs*22	LOVD: NF1_001526
190 (2)	1							
14 (1)	1	22(17)	SNV	2887C>T	2887C>T	Nonsense	Gln963*	ClinVar: 233495
109 (1)	1	22(17)	DEL	2948del	2948del	Frame-shift	Leu983Glnfs*9	New
19 (2)	1	22(17)	DEL	2970_2972del	2970_2972del	In-frame deletion	Met992del	LOVD: NF1_000277
134 (1)	1	23(18)	SNV	3040A>T	3040A>T	Nonsense	Lys1014*	ClinVar: 431616
140 (1)	4	23(18)	SNV	3104T>A	3104T>A	Missense	Met1035Lys	New
245 (1)	4	23(18)	SNV	3106A>G	3106A>G	Missense	Lys1036Glu	New
182 (1)	1	23(18)	SNV	3113+1G>A (splicing)	2991_3113del	Splicing	Tyr998_Arg1038del	LOVD: NF1_000306
230 (1)	3	25(19b)	SNV	3277G>A (cryptic splice site)	3275_3314del	Splicing	Gly1092Aspfs*7	LOVD: NF1_000340
74 (1)	1	26(20)	SNV	3326T>G	3326T>G	Nonsense	Leu1109*	New
41 (1)	4	26(20)	DEL	3347_3350del	3347_3350del	Frame-shift	Asp1116Alafs*25	LOVD: NF1_000351
255 (1)	1							
115 (1)	3	26(20)	SNV	3445A>G	3445A>G	Missense	Met1149Val	LOVD: NF1_000356
215 (1)	3							
20 (2)	1	26(20)	SNV	3496+1G>A	3315_3496del	Splicing	Tyr1106Leufs*28	HGMD: CS072245
40 (2)	5	27(21)	DEL	3502_3519del	3502_3519del	In-frame deletion	Gly1168_Leu1173del	New
284 (2)	6	27(21)	SNV	3592G>A	3592G>A	Missense	Glu1198Lys	New
256 (1)	1	27(21)	SNV	3610C>G	3610C>G	Missense	Arg1204Gly	LOVD: NF1_000372
104 (1)	1	28(22)	SNV	3826C>T	3826C>T	Nonsense	Arg1276*	LOVD: NF1_000403
108 (1)	1							
166 (1)	1							
225 (1)	1	28-29	DEL	(3708+1_3709-1)_(3973+1_3974-1)del (intragenic deletion ex. 28-29)	not determined	Intragenic deletion	?	New
275 (1)	3	29(23)	DEL	3899del	3899del	Frame-shift	Leu1300Profs*9	New
180 (1)	1	29(23)	SNV	3916C>T	3916C>T	Nonsense	Arg1306*	LOVD: NF1_000416
58 (1)	1	29(23)	DEL	3972del	3972del	Frame-shift	Arg1325Glyfs*2	New
247 (1)	1	29(23)	SNV	3974G>A	3873_3976del	Splicing	Tyr1292Argfs*7	LOVD: NF1_001992
129 (1)	1	30(23-1)	INS	4100_4103dup	4100_4103dup	Frame-shift	Tyr1369Phefs*6	New
36 (1)	2	32(24)	DEL	4168del	4168del	Frame-shift	Leu1390Serfs*17	LOVD: NF1_000458
162 (1)	1	32(24)	SNV	4172G>C	4172G>C	Missense	Arg1391Thr	LOVD: NF1_000461
285 (1)	1	32(24)	SNV	4269+2T>C	not determined	Splicing	?	New
261 (1)	5	33(25)	SNV	4276C>G	4276C>G	Missense	Gln1426Glu	LOVD: NF1_001275
154 (1)	1	33(25)	SNV	4278G>C	4278G>C	Missense	Gln1426His	LOVD: NF1_000483
32 (4)	5	35(27a)	SNV	4515-21T>G (splicing)	4514_4515insTTTGCTGTATCTAG	Splicing	Arg1505Serfs*53	New
16 (13)	1	35(27a)	SNV	4515-2A>G	4514_4515insTTTGCTGTATCTGG	Splicing	Arg1505Serfs*53	LOVD: NF1_000518
28 (1)	1	35(27a)	SNV	4537C>T	4537C>T	Nonsense	Arg1513*	LOVD: NF1_000521
9 (2)	1	35(27a)	SNV	4637C>A	4637C>A	Nonsense	Ser1546*	LOVD: NF1_000534
181 (1)	1	35(27a)	DEL	4644del	4644del	Frame-shift	Phe1548Leufs*5	New
66 (1)	1	36(27b)	DEL	4680_4683del	4680_4683del	Frame-shift	Glu1561Asnfs*5	New
173 (1)	4	36(27b)	DEL	4691del	4691del	Frame-shift	Lys1564Argfs*3	New
70 (1)	4	36(27b)	SNV	4768C>T	4768C>T	Missense	Arg1590Trp	HGMD: CM971051
4 (1)	1	37(28)	SNV	4780del	4780del	Frame-shift	Thr1594Leufs*9	New
119 (1)	2	37(28)	DEL	4840_4854del	4840_4854del	In-frame deletion	Tyr1614_Tyr1618del	LOVD: NF1_001657
89 (1)	1	37(28)	DEL	4914_4917del	4914_4917del	Frame-shift	Lys1640Glyfs*36	LOVD: NF1_000586
193 (1)	1	37(28)	SNV	4922G>A	4922G>A	Nonsense	Trp1641*	LOVD: NF1_001303
80 (2)	1	37(28)	DEL	4973_4978del	4973_4978del	In-frame deletion	Ile1658_Tyr1659del	LOVD: NF1_000597
95 (2)	4	37-51	DUP	5035-?_7426-?dup (intragenic duplication ex. 37-51)	not determined	Intragenic duplication	?	New
10 (2)	1	38(29)	SNV	5264C>G	5264C>G	Nonsense	Ser1755*	HGMD: CM001260
1 (2)	4	38(29)	SNV	5401C>T	5401C>T	Nonsense	Gln1801*	LOVD: NF1_001390
101 (2)	5	38(29)	SNV	5425C>T	5425C>T	Missense	Arg1809Cys	LOVD: NF1_000653
112 (5)	5							
178 (2)	5							
302 (1)	3							
155 (1)	4	38(29)	SNV	5426G>C	5426G>C	Missense	Arg1809Pro	ClinVar: 208855
124 (3)	5	38(29)	SNV	5426G>T	5426G>T	Missense	Arg1809Leu	LOVD: NF1_000654
156 (1)	4							
229 (1)	5							
175 (1)	3	38(29)	SNV	5437T>C	5437T>C	Missense	Ser1813Pro	New
164 (2)	1	38(29)	SNV	5483A>T	5483A>T	Missense	Asp1828Val	LOVD: NF1_000666
259 (1)	1	38(29)	SNV	5543T>A	5543T>A	Nonsense	Leu1848*	LOVD: NF1_000670
163 (1)	1	39(30)	DEL	5592_5596del	5592_5596del	Frame-shift	Asn1864Lysfs*26	New
231 (1)	3	39(30)	SNV	5608C>T	5608C>T	Nonsense	Gln1870*	ClinVar: 237577
22 (1)	1	39(30)	SNV	5676G>T	5676G>T	Missense	Lys1892Asn	New
177 (3)	1	39(30)	SNV	5719G>T	5719G>T	Nonsense	Glu1907*	ClinVar: 187652
26 (2)	1	39(30)	DEL	5739del	5739del	Frame-shift	Phe1913Leufs*8	New
24 (2)	1	40(31)	SNV	5839C>T	5839C>T	Nonsense	Arg1947*	LOVD: NF1_000711
171 (1)	1	40(31)	SNV	5842C>T	5842C>T	Nonsense	Gln1948*	LOVD: NF1_001913
151 (1)	1	40(31)	SNV	5928G>A	5928G>A	Nonsense	Trp1976*	LOVD: NF1_002495
71 (1)	4	40(31)	SNV	5938G>C	5938G>C	Missense	Gly1980Arg	ClinVar: 457773
282 (1)	3	41(32)	SNV	5944-1G>C (cryptic splice site)	5946_5952del	Splicing	Thr1983Cysfs*6	ClinVar: 431977
135 (1)	3	41(32)	SNV	5944-5A>G (cryptic splice site)	5943_5944insCTAG	Splicing	Ile1982Leufs*7	LOVD: NF1_001321
34 (2)	1	42(33)	SNV	6085-2A>T (splicing)	6085_6364del	Splicing	Val2029Lysfs*7	LOVD: NF1_001919
83 (1)	1	42(33)	SNV	6243C>A	6243C>A	Nonsense	Y2081*	New
223 (1)	1	42(33)	SNV	6335T>C	6335T>C	Missense	Leu2112Pro	LOVD: NF1_000756
233 (1)	1	42(33)	SNV	6364+4A>G	6085_6364del	Splicing	Val2029Lysfs*7	HGMD: CS941517
97 (1)	3	43(34)	SNV	6579+1G>T (splicing)	6365_6579del	Splicing	Glu2122Glyfs*27	LOVD: NF1_000784
253 (1)	3	43(34)	SNV	6579+2T>C	not determined	Splicing	?	New
42 (1)	3	44(35)	SNV	6606C>A	6606C>A	Nonsense	Cys2202*	LOVD: NF1_001338
137 (1)	1	44(35)	SNV	6611G>A	6611G>A	Nonsense	Trp2204*	LOVD: NF1_001584
170 (1)	3	44(35)	SNV	6641+1G>C	6580_6641del	Splicing	Ala2194Ilefs*6	LOVD: NF1_000796
139 (1)	1	45(36)	DEL	6688del	6688del	Frame-shift	Val2230Serfs*14	LOVD: NF1_001670
50 (1)	1	45(36)	SNV	6709C>T	6709C>T	Nonsense	Arg2237*	LOVD: NF1_000802
73 (1)	1	46(37)	INS	6791_6792insAA	6791_6792insAA	Frame-shift	Tyr2264*	LOVD: NF1_001349
51 (2)	1	46(37)	INS	6791dup	6791dup	Frame-shift	Tyr2264*	LOVD: NF1_000815
118 (1)	3	46(37)	SNV	6792C>A (STOP determining splicing)	6757_6858del	Splicing	Ala2253_Lys2286del	LOVD: NF1_000816
143 (1)	4							
176 (1)	1							
299 (1)	3	46(37)	SNV	6792C>G (STOP determining splicing)	6757_6858del	Splicing	Ala2253_Lys2286del	LOVD: NF1_000817
30 (1)	1	46(37)	SNV	6858+1G>T (splicing)	6757_6858del	Splicing	Ala2253_Lys2286del	LOVD: NF1_000824
268 (1)	1	46(37)	SNV	6858+2T>C	6757_6858del	Splicing	Ala2253_Lys2286del	HGMD: CS073509
47 (1)	3	47(38)	DEL	6881del	6881del	Frame-shift	Leu2294Profs*4	LOVD: NF1_001726
84 (1)	1	47(38)	DEL	6898_6903del	6898_6903del	In-frame deletion	Ala2300_Val2301del	New
88 (2)	1	47(38)	SNV	6955C>T	6955C>T	Nonsense	Gln2319*	New
276 (1)	1	47(38)	DEL	6974_6977del	6974_6977del	Frame-shift	Asp2325Valfs*49	LOVD: NF1_001352
94 (1)	1	48(39)	INS	7089dup	7089dup	Frame-shift	Asn2364*	LOVD: NF1_001359
183 (1)	1							
43 (3)	1	48(39)	DEL	7125del	7125del	Frame-shift	Tyr2377Thrfs*20	LOVD: NF1_000849
158 (3)	1							
169 (1)	1	49(40)	DEL	7169_7170del	7169_7170del	Frame-shift	Arg2390Asnfs*10	New
79 (2)	1	49(40)	SNV	7184T>C	7184T>C	Missense	Leu2395Pro	LOVD: NF1_000857
227 (1)	1	49(40)	INS	7232dup	7232dup	Frame-shift	Asn2411Lysfs*16	New
185 (1)	1	50(41)	SNV	7259C>A	7259C>A	Missense	Ala2420Asp	LOVD: NF1_000867
3 (1)	1	50(41)	SNV	7285C>T	7285C>T	Nonsense	Arg2429*	LOVD: NF1_000871
87 (1)	1							
37 (1)	3	51(42)	DEL	7518del	7518del	Frame-shift	Gln2507Asnfs*20	ClinVar: 237598
130 (1)	4	52(43)	SNV	not determined	7553_7675del	In-frame deletion	Gly2518_Met2558del	
12 (2)	1	53(44)	DEL	7686del	7686del	Frame-shift	Ile2563Phefs*40	LOVD: NF1_002529
25 (2)	1	54(45)	INS	7874_7875dup	7874_7875dup	Frame-shift	Ser2626Profs*33	New
103 (1)	1	56(47)	SNV	8051-1G>C (splicing)	8051_8097del	Splicing	Ser2684Thrfs*9	New
260 (1)	4	57(48)	INS	8207_8231dup	8207_8231dup	Frame-shift	Leu2745Serfs*14	New
61 (1)	2	all	DEL	-718-?_8375+?del (Microdeletion 17q11.2)	Microdeletion 17q11.2	Microdeletion 17q11.2	?	LOVD: NF1_000001
64 (1)	2							
69 (1)	2							
77 (1)	2							
78 (1)	1							
99 (1)	2							
127 (1)	2							
136 (1)	1							
211 (1)	1							
277 (1)	1							

^1^ Number of family members presenting the variant is reported in parentheses. ^2^ Type of variant: SNV = Single-nucleotide variant, DEL = Deletion, DUP = Duplication, INS = Insertion, INDEL = Insertion-deletion, DIM = Deep intronic mutation. ^3^ ID of annotated variants in ClinVar (www.ncbi.nlm.nih.gov/clinvar), Human Genome Variation Database (HGMD; www.hgmd.cf.ac.uk), and Leiden Open Variation Database (LOVD; databases.lovd.nl/shared/genes/NF1).

**Table A2:** Unique variants identified in *SPRED1* (RefSeq NM_152594.2).

Family ID ^1^	Group	Exon	Type ^2^	Genomic	cDNA	Effect	Protein	ClinVar/HGMD/LOVD ID ^3^
11(10)	5	2	DEL	49_53del	49_53del	Frame-shift	Val17Serfs*8	New
224(1)	3	2	SNV	52C>T	52C>T	Nonsense	Arg18*	LOVD: SPRED1_000177
92(3)	5	2	SNV	70C>T	70C>T	Nonsense	Arg24*	LOVD: SPRED1_000014
179(3)	5	2	SNV	74A>G	74A>G	Missense	Asp25Gly	ClinVar: 391600
161(2)	5	3	SNV	229A>T	229A>T	Nonsense	Lys77*	LOVD: SPRED1_000121
157(1)	4	6–7	DEL	618-?_*91+?del (intragenic deletion ex. 6-7)	?	?	?	New
167(4)	5	7	SNV	973C>T	973C>T	Nonsense	Arg325*	LOVD: SPRED1_000077
286(2)	5	7	DUP	993dup	993dup	Frame-shift	Arg332Thrfs*12	New

^1^ Number of family members presenting the variant is reported in parentheses. ^2^ Type of variant: SNV = Single-nucleotide variant, DEL = Deletion, DUP = Duplication. ^3^ ID of annotated variants in ClinVar (www.ncbi.nlm.nih.gov/clinvar), Human Genome Variation Database (HGMD; www.hgmd.cf.ac.uk), and Leiden Open Variation Database (LOVD; databases.lovd.nl/shared/genes/SPRED1).

**Table A3:** Clinical features of 245 probands with suspicion of NF1 or an NF1-like condition (the most serious clinical features that could reduce patients’ life expectancy are highlighted in bold).

Group	Family ID	Patient ID	Sex	Molecularly Characterized Affected Relatives	Sporadic (Y/N)	Paternal/Maternal Inheritance	Age (yy:mm)		CALMs (≥6)		Freckling		Lisch Nodules		OPG		Bone Dysplasia		Neurofibromas (Cutaneous or Plexiform)		Other Clinical Features ^1^
							T0	T1	T0	T1	T0	T1	T0	T1	T0	T1	T0	T1	T0	T1	
1	2	4	F	0	N	Maternal	2	12:06	+	+	-	+	+	+	-	-	-	-	+	+	**LGG**, DLL
1	3	5	M	0	N	Maternal	6:08	14	+	+	+	+	-	-	-	-	-	-	-	+	Thor
1	4	6	M	0	N	Paternal	14:02	18:05	+	+	+	+	-	-	n.a.	n.a.	-	-	-	-	
1	6	8	M	2	N	Maternal	6	13	+	+	+	+	+	+	-	-	-	-	+	+	DLL, DS, ID
1	7	10	M	1	N	Paternal	20	22	+	+	+	+	+	+	-	-	-	-	-	-	NBOs, Epy
1	8	12	M	0	N	Paternal	10	19:08	+	+	+	+	+	+	-	-	-	-	+	+	DLL
1	9	13	F	1	N	Paternal	14:09	17	+	+	+	+	+	+	-	-	-	-	+	+	UH
1	10	16	F	1	N	Maternal	10	15:04	+	+	-	+	-	-	+	+	-	-	+	+	NBOs
1	12	116	M	3 (2 twin)	N	Paternal	10	14:08	+	+	-	+	-	-	-	-	-	-	-	-	LD, SP
1	14	23	M	0	Y		9:09	11:08	+	+	+	+	+	+	-	-	-	-	-	-	
1	15	24	M	0	N	Maternal	10	11	+	+	+	+	+	+	-	-	-	-	-	-	DS
1	16	26	M	12	N	Maternal	10:01	12:04	+	+	+	+	+	+	-	-	-	-	+	+	UH, **MMS**, Thor, NBOs, DLL, PmD, LD
1	17	37	F	1	N	Paternal	3:01	9:01	+	+	-	+	-	-	-	-	-	-	-	+	LD, NBOs
1	18	38	F	0	N	Paternal	5:09	8:11	+	+	+	+	-	-	-	-	-	-	+	+	Noon, MacroC, SP, Thor
1	19	40	F	1	N	Maternal	12:01	15:08	+	+	+	+	+	+	-	-	-	-	-	-	Noon, MacroC, SP, Thor, SS, BvP, BP,
1	20	41	M	1	N	Maternal	11	19	+	+	n.a.	n.a.	+	+	-	-	-	-	-	-	Thor
1	21	43	M	0	N	Paternal	21:11	22:08	+	+	+	+	+	+	-	-	-	-	+	+	
1	22	45	M	0	N	Maternal	14:08	15:06	+	+	+	+	+	+	-	-	-	-	+	+	
1	23	48	F	1 (twin)	Y		15	17:06	+	+	+	+	+	+	-	-	-	-	+	+	DS, DE, VS, LD, SP
1	24	50	M	1	N	Maternal	13:09	17:06	+	+	+	+	n.a.	n.a.	+	+	-	-	+	+	SS, DE, DS, NBOs
1	25	51	F	1	N	Maternal	11:02	11:07	+	+	+	+	+	+	-	-	-	-	-	-	
1	26	53	F	1	N	Maternal	6:01	10	+	+	-	+	-	-	-	-	n.a.	n.a.	-	-	LD
1	27	54	M	1 (twin)	Y		15:03	18:11	+	+	+	+	+	+	-	-	-	-	-	+	Thor
1	28	56	F	0	N	Maternal	7	8:11	+	+	+	+	n.a.	n.a.	-	-	-	-	+	+	
1	29	58	F	1	N	Paternal	19:04	22:11	+	+	+	+	+	+	-	-	-	-	+	+	DS, NBOs
1	30	61	M	0	N	Paternal	11:09	13:01	+	+	+	+	-	n.a.	-	-	-	-	-	-	Thor
1	31	62	M	0	N	Paternal	9:07	10:03	+	+	+	+	+	+	+	+	-	-	-	-	DLL, NBOs
1	33	65	F	1	N	Paternal	14:02	14:08	+	+	+	+	+	+	n.a.	n.a.	-	-	-	-	SS, DLL, MacroC, DS, Noon
1	34	66	M	1	N	Maternal	14:05	16	+	+	+	+	+	+	-	-	-	-	-	-	**MMS**
1	35	67	F	1	N	Paternal	22	27	+	+	+	+	-	-	-	-	-	-	+	+	
1	38	72	F	0	N	Paternal	6:04	7:05	+	+	-	+	-	-	-	-	-	-	-	-	NA, MacroC
1	39	76	M	1	N	Maternal	8:06	9:05	+	+	+	+	n.a.	n.a.	n.a.	n.a.	-	-	-	-	BP
1	43	88	F	2	N	Maternal	7	8:01	+	+	+	+	-	-	+	+	-	-	+	+	**Leuk**, DLL, MacroC, NBOs
1	44	91	M	2	N	n.a.	35	36	+	+	+	+	-	-	n.a.	n.a.	-	-	+	+	
1	46	93	M	0	Y		14	18	+	+	+	+	-	+	-	-	-	+	+	+	Thor, DS
1	48	99	F	0	Y		20:01	21:09	+	+	+	+	+	+	-	-	-	-	+	+	
1	50	101	M	0	Y		4	6:06	+	+	-	+	+	+	-	+	-	-	+	+	SS, NBOs
1	51	102	F	1	N	Maternal	5	7:08	+	+	+	+	+	+	-	-	-	-	+	+	
1	52	104	F	0	Y		13	14:02	+	+	+	+	+	+	-	-	-	-	-	-	BvP, NA, LD, SP, **CD**
1	55	107	M	1 (twin)	Y		9:02	14:04	+	+	+	+	+	+	-	+	-	-	+	+	DLL, NBOs
1	58	112	F	0	N	Maternal	7	10	+	+	+	+	+	+	n.a.	n.a.	-	-	+	+	mild ID
1	63	125	F	0	Y		16:04	16:07	+	+	+	+	+	+	-	-	-	-	+	+	DLL, NBOs, Thor, MacroC
1	65	128	F	0	Y		12	19:02	+	+	+	+	+	+	-	-	-	-	+	+	LD, DS, NBOs, Thor
1	66	129	M	0	Y		7	13:03	+	+	+	+	-	-	-	-	-	-	+	+	
1	67	130	M	0	Y		12	13:03	+	+	+	+	n.a.	n.a.	-	-	-	-	+	+	
1	68	132	M	0	Y		7	13:06	+	+	-	-	+	+	-	-	-	-	+	+	DLL, NBOs
1	72	137	M	0	Y		21	22:02	+	+	+	+	+	+	-	-	+	+	-	-	
1	73	138	F	0	Y		17:03	19:05	+	+	+	+	-	-	-	-	-	-	+	+	MacroC
1	74	139	F	0	Y		12:04	13:04	+	+	+	+	+	+	-	-	-	-	+	+	NBOs, LD, SP
1	75	140	M	0	N	Maternal	6:05	8:02	+	+	+	+	-	-	-	-	-	-	-	-	Thor, LD, NBOs
1	76	141	F	0	Y		9:09	12:03	+	+	+	+	+	+	-	-	-	-	+	+	DLL
1	78	149	F	0	Y		16	18	+	+	n.a.	+	-	+	-	-	-	-	+	+	
1	79	151	M	1	N	Maternal	5:06	8:05	+	+	+	+	-	+	-	+	-	-	+	+	NBOs, CIm, SP, DS, Cry, Noon
1	80	155	M	1	N	Paternal	4:03	5:05	+	+	-	+	-	-	-	-	-	-	-	-	NBOs, ID
1	82	158	M	0	N	Maternal	4:11	5:11	+	+	-	-	-	+	-	-	-	-	-	-	DLL
1	83	160	F	0	Y		8	9:07	+	+	+	+	-	+	-	-	-	-	+	+	NBOs, BvP
1	84	161	F	0	Y		23:01	25:11	+	+	+	+	+	+	-	-	-	--	+	+	NBOs
1	85	162	F	0	N	Paternal	1:11	3:01	+	+	+	+	-	-	-	-	-	-	-	-	MacroC, UH
1	87	171	F	0	N	Paternal	7	8:01	+	+	+	+	-	+	-	-	-	-	+	+	DS, DE, VS, LD, SP, DLL,
1	88	253	F	1	N	Maternal	7:06	8:06	+	+	+	+	+	+	-	-	-	-	-	-	NBOs
1	89	175	F	0	Y		20	21	+	+	+	+	+	+	-	-	-	-	-	-	LD, NBOs
1	93	185	M	2	N	Maternal	7:06	11	+	+	+	+	+	+	-	-	-	-	+	+	PmD, SP
1	94	187	M	0	N	Maternal	15	18:04	+	+	+	+	-	-	-	-	-	-	-	-	BvP
1	98	204	F	0	N	Paternal	13:06	16:06	+	+	+	+	+	+	-	+	-	-	+	+	DS
1	102	218	F	1	N	Maternal	25	27	+	+	+	+	-	-	-	-	-	-	-	-	Myo, MLyn
1	103	220	F	0	Y		21	23	+	+	-	-	+	+	-	-	-	-	+	+	SF
1	104	223	F	0	Y		15	16:05	+	+	+	+	n.a.	n.a.	-	-	-	-	+	+	NBOs, BvP, MacroC
1	108	228	M	0	Y		0:05	3:05	-	+	-	+	-	-	-	-	-	-	+	+	NA, MacroC, Thor, PmD
1	109	236	M	1	N	Maternal	12:07	13:01	+	+	+	+	+	+	-	-	-	-	+	+	
1	114	247	M	0	Y		18	21:08	+	+	-	-	+	+	-	-	-	-	-	-	
1	116	250	M	0	Y		12:03	13:03	+	+	+	+	-	-	-	-	-	-	+	+	
1	126	271	M	0	N	Maternal	4:05	5:03	+	+	-	-	+	+	+	+	-	-	+	+	UH, NBOs, BvP, LD
1	129	276	M	0	Y		6:04	7	+	+	+	+	-	-	-	-	-	-	+	+	PmD, DLL, Epy
1	132	286	M	1	N	Paternal	11:09	12:01	+	+	+	+	+	+	+	+	-	-	-	-	NBOs, SP, PmD
1	133	287	F	1	N	n.a.	1	1:06	+	+	+	+	+	+	-	-	-	-	-	-	Noon
1	134	289	M	0	Y		10	10:08	+	+	+	+	+	+	-	-	-	-	-	-	
1	136	291	M	0	Y		15:05	15:09	+	+	+	+	n.a.	n.a.	-	-	-	-	+	+	LD, PmD, SD, NBOs, BvP, SF
1	137	309	F	0	Y		0:08	1:01	+	+	-	-	-	-	-	-	-	-	+	+	
1	139	301	M	1	N	n.a.	30:06	31:02	+	+	-	-	+	+	-	-	-	-	+	+	
1	144	317	F	2	N	Maternal	30	30:03	+	+	+	+	+	+	-	-	-	-	+	+	MacroC, Noon
1	150	325	M	0	Y		11:11	13	+	+	+	+	+	+	-	-	-	-	+	+	BSL, LD
1	151	326	F	0	Y		14:01	14:08	+	+	+	+	+	+	+	+	-	-	-	-	**MMS**
1	152	328	M	0	Y		13	14:06	+	+	+	+	+	+	+	+	-	-	-	-	
1	153	329	M	0	Y		19	19:6	+	+	+	+	+	+	-	-	-	-	-	-	
1	154	331	M	0	Y		20	20:06	+	+	+	+	+	+	-	-	-	-	+	+	
1	158	341	F	2	N	Maternal	15:03	15:09	+	+	+	+	+	+	-	-	-	-	-	-	
1	159	343	M	0	Y		14:11	15:05	+	+	+	+	+	+	+	+	-	-	-	-	
1	160	385	M	0	Y		29:01	30	+	+	-	-	+	+	-	-	-	-	-	-	PS, **CD**, MacroC, Noon
1	162	354	F	0	Y		42:06	43	+	+	+	+	n.a.	n.a.	-	-	-	-	+	+	PS, SS, BvP
1	163	350	F	0	Y		49:01	50:08	+	+	+	+	+	+	-	-	-	-	-	-	
1	164	384	F	1	N	Paternal	12:06	13:04	+	+	+	+	+	+	-	-	-	-	-	-	NBOs, **MMS**
1	165	52	F	0	N	Paternal	20	26	+	+	+	+	+	+	-	-	-	-	+	+	
1	166	28	F	0	N	Maternal	17:01	19	+	+	+	+	+	+	-	-	-	-	+	+	HY
1	169	334	M	0	Y		18	19	+	+	+	+	+	+	-	-	-	-	+	+	Thor, MacroC
1	171	405	M	0	Y		5:05	6:01	+	+	-	-	+	+	+	+	-	-	+	+	
1	172	395	M	0	Y		37:02	37:08	+	+	+	+	+	+	-	-	-	-	-	-	
1	174	435	F	0	Y		60:02	60:09	+	+	+	+	-	-	-	-	-	-	+	+	
1	176	463	F	0	Y		37	37:03	+	+	+	+	+	+	-	-	-	-	+	+	MacroC
1	177	199	M	2	N	Paternal	10:03	13:09	+	+	+	+	+	+	n.a.	n.a.	-	-	-	-	PmD, SP, Cry
1	180	340	M	0	Y		13	15	+	+	+	+	+	+	-	-	-	-	-	-	
1	181	450	F	0	Y		15:01	16:03	+	+	+	+	+	+	-	-	-	-	-	-	
1	182	95	M	0	Y		20	21	+	+	n.a.	n.a.	n.a.	n.a.	-	-	-	-	+	+	NBOs
1	183	146	M	0	Y	Paternal	15	17	+	+	+	+	-	-	n.a.	n.a.	-	-	-	-	HY
1	184	166	M	1	N	Maternal	24	31	+	+	n.a.	n.a.	+	+	-	-	-	-	+	+	NBOs, LD, DLL, DS, PmD, ID, VS
1	185	146	M	0	Y		69	76	+	+	n.a.	n.a.	+	+	-	-	-	-	+	+	
1	186	189	F	0	Y		28	34	+	+	+	+	+	+	-	-	-	-	+	+	NBOs, LD, SP, DS, PmD, ID, BvP, SS, Epy, AD
1	188	255	M	0	Y		41	42	+	+	+	+	n.a.	n.a.	+	-	-	-	+	+	**LGG**, **Malignancies**, SS
1	190	367	F	1	N	Maternal	68	70	+	+	+	+	n.a.	n.a.	-	-	-	-	+	+	DS
1	192	425	M	0	Y		20	22	+	+	+	+	+	+	+	+	-	-	+	+	NBOs, LD, DLL, DS, MacroC, HY
1	193	431	M	0	Y		31	36	+	+	+	+	+	+	-	-	-	-	+	+	NBOs, LD, SP, DLL, PmD, **Malignancies**, HY
1	195	86	F	0	Y		3	20	+	+	+	+	-	-	-	-	-	-	-	-	
1	209	345	M	0	Y		4	6	+	+	+	+	+	+	-	-	-	-	-	-	
1	211	432	M	0	Y		33	33	+	+	+	+	+	+	-	-	-	-	+	+	
1	212	442	M	0	Y		34	36:8	+	+	+	+	n.a.	n.a.	n.a.	n.a.	-	-	-	-	Dlip
1	214	453	F	0	Y		13	14:5	+	+	+	+	+	+	n.a.	n.a.	-	-	+	+	BvP
1	217	470	F	0	Y		20	21	+	+	+	+	+	+	+	+	-	-	+	+	NBOs
1	218	471	M	0	Y		7	7:09	+	+	-	-	-	-	+	+	-	-	+	+	NBOs, ID, SP, Noon
1	220	480	F	0	Y		19	19	+	+	+	+	-	-	-	-	-	-	+	+	NBOS, DLL, DS, SS
1	221	481	M	0	Y		9	10:02	+	+	+	+	-	-	n.a.	-	+	+	-	-	NBOs, ID, Bover
1	222	483	M	0	N		0:08	1:06	+	+	-	-	-	-	n.a.	n.a.	-	-	-	-	**CD**
1	223	491	M	0	Y		37	40	+	+	+	+	-	-	-	-	-	-	+	+	DS, SF
1	225	495	F	0	Y		8	10:01	+	+	+	+	-	-	-	-	-	-	-	-	DE, VS, Noon, SS, NBOs
1	227	500	M	0	Y		13	13	+	+	+	+	+	+	+	+	-	-	+	+	
1	233	523	F	0	Y		21	21:05	+	+	-	-	-	-	-	-	-	-	+	+	
1	234	524	M	1	N	Paternal	17	18:2	+	+	+	+	-	-	-	-	-	-	+	+	**Hyd**, NA, Noon, Chem, SS
1	241	548	M	0	Y		30	33	+	+	+	+	-	-	-	-	-	-	+	+	NBOs, LD, DS, BP
1	247	567	M	0	N	Maternal	5	7	+	+	+	+	-	-	+	+	-	-	+	+	NBOs
1	251	575	M	0	Y		10	11	+	+	+	+	-	-	+	+	-	-	+	+	NBOs
1	255	585	M	0	Y		12	13:03	+	+	-	-	-	-	-	-	-	-	-	-	ID, NBOs
1	256	592	M	0	Y		19	20	+	+	+	+	-	-	-	-	-	-	-	-	
1	259	602	M	0	Y		13	14:25	+	+	+	+	-	-	-	-	+	+	+	+	AD
1	268	632	M	0	Y		15	17	+	+	-	-	-	-	-	-	-	-	+	+	
1	276	646	M	0	Y		1	30:05	+	+	-	-	-	-	+	+	-	-	-	-	
1	277	647	M	0	Y		46	48	+	+	+	+	-	-	-	-	-	-	+	+	DLL, DS
1	285	657	M	0	Y		51	54	+	+	-	-	-	-	-	-	-	-	+	+	HY, SF
1	288	665	M	0	Y		5	6	+	+	+	+	-	-	n.a.	n.a.	-	-	+	+	DD, SP
1	289	667	F	0	Y		55	58	+	+	+	+	+	+	-	-	-	-	+	+	HY, **Malignancies**
1	292	670	M	0	Y		11	11:04	+	+	+	+	n.a.	n.a.	-	-	-	-	+	+	NBOs, SP, LD
1	300	681	M	0	Y		45	47	+	+	+	+	+	+	-	-	-	-	+	+	NBOs, DS
2	36	70	M	0	N	Maternal	10	12:11	+	+	+	+	+	+	+	+	-	-	+	+	**RMS**, NBOs, MacroC, DS, VS
2	61	118	M	0	Y		2:01	3:11	+	+	-	+	-	-	-	-	-	-	+	+	ID, BP, MacroC, UH, Noon, Thor,
2	64	127	M	0	Y		14:06	16:02	+	+	+	+	+	+	-	-	-	-	+	+	ID, Thor
2	69	134	M	0	Y		16	17:02	+	+	+	+	+	+	-	-	-	-	+	+	Thor, NBOs, DLL, ID
2	77	147	M	0	Y		4:09	6:06	+	+	-	-	-	-	-	-	-	-	+	+	ID, BvP, NBOs, Thor,
2	99	205	F	0	Y		29	29:08	+	+	+	+	+	+	-	-	-	-	-	-	ID, NBOs, **MPNST**
2	107	227	M	0	Y		5:03	7	+	+	+	+	+	+	-	-	-	-	-	-	ID, SP, Noon, NA
2	119	259	M	0	Y		11:02	13	+	+	+	+	+	+	-	-	-	-	+	+	DE, DS, VS, ID, SP, LD, Noon, macroC, NBOs, DLL, BSL
2	127	272	M	0	Y		15	15:09	+	+	+	+	-	-	-	-	-	-	+	+	ID, PS, Epy
2	149	324	M	0	Y		14	16	+	+	+	+	-	-	+	+	-	-	+	+	ID
2	168	97	M	0	N	Paternal	20	23	+	+	+	+	+	+	+	+	-	-	+	+	ID
3	37	71	M	0	Y		9	12:11	+	+	+	+	-	-	-	-	-	-	-	-	**Leuk**
3	42	83	M	0	Y		6	8:09	+	+	-	-	-	+	-	-	-	-	-	-	
3	45	92	F	0	Y		9:02	14:08	+	+	-	+	-	-	-	-	-	-	-	+	Thor, DS, DLL
3	47	96	F	0	Y		4:11	5:11	+	+	+	+	-	+	-	-	-	-	-	-	
3	49	100	M	0	Y		1:04	3:01	+	+	-	+	-	-	-	-	-	-	-	+	BP, XG
3	53	105	M	0	Y		8	10	+	+	-	+	-	-	-	-	-	-	-	-	LD
3	57	110	F	0	Y		3:09	4:03	+	+	-	+	-	-	-	-	-	-	-	-	
3	81	157	M	0	Y		2:01	4:11	+	+	-	+	-	-	-	-	-	-	-	-	SP, Cry, NA, Thor, Noon
3	91	180	M	0	Y		3:05	4:07	+	+	-	-	-	-	-	-	-	-	-	-	DD, MacroC, SP
3	97	203	F	0	Y		3	7:2	+	+	-	+	-	-	-	-	-	-	-	+	
3	110	238	F	0	Y		1:01	4:02	+	+	+	+	-	-	-	-	-	-	-	-	
3	113	245	F	0	Y		9:01	12:02	+	+	-	-	-	-	-	-	-	-	-	-	
3	115	249	M	0	Y		9	9:02	+	+	+	+	-	-	-	-	-	-	-	-	DLL
3	118	257	M	0	Y		5:08	8	+	+	-	+	-	-	-	-	-	-	-	-	XG
3	120	260	F	0	Y		8:08	12	+	+	-	-	-	-	-	-	-	-	-	-	
3	121	261	F	0	Y		1:09	3:05	+	+	+	+	-	-	-	-	-	-	-	-	
3	125	268	F	0	Y		3:05	3:06	+	+	+	+	-	-	-	-	-	-	-	-	UH, XG
3	128	273	M	0	Y		8:08	10:04	+	+	-	-	-	-	-	-	-	-	-	-	ID, UH, Thor
3	131	282	F	0	Y		4	6	+	+	-	-	-	-	n.a.	n.a.	-	-	-	-	NA
3	135	290	F	0	Y		9:03	10:01	+	+	+	+	-	-	-	-	-	-	-	-	
3	138	293	M	0	Y		5:08	5:01	+	+	+	+	-	-	-	-	-	-	-	-	
3	142	314	M	0	Y		8:06	10	+	+	-	-	-	-	-	-	-	-	-	-	
3	145	321	F	0	Y		9:01	10:01	+	+	-	-	-	-	-	-	-	-	-	-	
3	146	322	F	0	Y		6	7	+	+	-	-	-	-	-	-	-	-	-	-	
3	170	145	M	0	Y		3:05	6:02	+	+	+	+	-	-	-	-	-	-	-	-	MacroC, NBOs
3	175	358	F	0	Y		2:08	4:04	+	+	-	+	-	-	-	-	-	-	-	-	SS, SP
3	202	374	M	0	Y		8	9	+	+	-	-	-	-	-	-	-	-	-	-	Thor
3	215	464	F	0	Y		2	3:03	+	+	-	-	-	-	-	-	-	-	-	-	SP, **CD**
3	224	494	M	0	Y		9	12	+	+	-	-	-	-	-	-	-	-	-	-	LD, BvP
3	228	503	M	0	Y		2	3:08	+	+	-	-	-	-	n.a.	n.a.	-	-	-	-	
3	230	507	M	0	Y		7	8:03	+	+	-	-	-	-	n.a.	n.a.	-	-	-	-	
3	231	508	F	0	Y		1	1:03	+	+	-	-	-	-	n.a.	n.a.	-	-	-	-	
3	239	536	M	0	Y		5		+	+	-	-	-	-	n.a.	n.a.	-	-	-	-	MacroC
3	244	555	M	0	Y		7	8:01	+	+	-	-	-	-	-	-	-	-	-	-	
3	252	576	M	0	Y		3	5	+	+	+	+	-	-	-	+	-	-	-	+	ID, SP, NBOs, CIm, **MBG**, secondary Epy, DE, VS
3	253	579	M	0	Y		7	9	+	+	-	-	-	-	n.a.	n.a.	-	-	-	-	SP
3	275	644	M	0	Y		6	7:02	+	+	+	+	-	-	-	-	-	-	-	+	SP, DD
3	278	649	M	0	Y		3	3:08	+	+	-	-	-	-	n.a.	n.a.	-	-	-	-	NA
3	279	650	M	0	Y		2	3:25	+	+	-	-	-	-	n.a.	n.a.	-	-	-	-	AtC
3	282	653	M	0	Y		8	8:06	+	+	+	+	-	-	n.a.	n.a.	-	-	-	-	
3	294	672	F	0	Y		2	2:10	+	+	-	+	-	-	n.a.	n.a.	-	-	-	-	**CD**
3	297	677	F	0	Y		7	8	+	+	-	-	-	-	n.a.	n.a.	-	-	-	-	AtC
3	299	680	M	0	Y		6	8	+	+	-	-	-	+	-	-	-	-	-	-	ID
3	302	683	M	0	Y		1	2	+	+	-	-	-	-	-	-	-	-	-	-	
4	1	3	M	1 (twin)	Y		12	14	+	+	+	+	n.a.	n.a.	-	-	-	-	-	-	LD
4	5	7	M	0	Y		13:03	17	+	+	-	-	-	+	-	-	-	-	-	-	Thor
4	13	20	M	0	Y		10:07	12:08	+	+	-	-	-	+	-	-	-	-	-	-	ArC, NBOs, BP, Noon, Thor, SP, **MMS**
4	41	82	F	0	Y		16	18	+	+	+	+	-	-	-	-	-	-	-	-	
4	54	106	M	0	Y		12:03	13:01	+	+	+	+	-	-	-	-	-	-	-	-	UH, NBOs
4	56	109	M	0	Y		15:04	16:02	+	+	-	-	-	-	-	-	-	-	-	-	
4	59	113	F	0	Y		11:03	13:05	+	+	-	+	-	-	-	-	-	-	-	-	DS, SS
4	70	135	M	0	Y		17:06	18	+	+	-	-	-	-	n.a.	n.a.	-	-	-	-	SS, BvP
4	71	136	M	0	Y		15:07	17:05	+	+	-	-	-	-	-	-	-	-	-	-	ID, SP, DLL
4	86	163	F	0	Y		14:02	17:01	+	+	-	-	-	-	-	-	-	-	-	-	LD
4	95	198	F	1	Y		13:05	15:03	+	+	-	+	-	-	-	-	-	-	-	+	
4	96	202	F	0	Y		14:01	15	+	+	+	+	-	-	-	-	-	-	-	-	
4	106	225	M	0	Y		10:06	12:09	+	+	+	+	-	-	-	-	-	-	-	-	
4	117	251	M	0	Y		13:04	14:04	+	+	-	-	-	-	-	-	-	-	-	-	Myo, AtIN
4	123	264	M	0	Y		11	14:07	+	+	-	-	-	-	-	-	-	-	-	-	
4	130	278	M	0	Y		16:03	18:01	+	+	+	+	-	-	-	-	-	-	-	-	
4	140	306	F	0	Y		16:04	17	+	+	+	+	-	-	-	-	-	-	-	-	DS, BvP, DLL
4	143	315	F	0	Y		14:01	14:08	+	+	+	+	-	+	-	-	-	-	-	-	
4	155	339	F	0	Y		11:09	12:04	+	+	-	-	-	-	n.a.	n.a.	-	-	-	-	MacroC, LD
4	156	195	F	0	Y		11:03	13:09	+	+	-	-	-	-	-	-	-	-	-	-	
4	157	196	F	0	Y		11:09	14:08	+	+	-	+	-	-	-	-	-	-	-	-	
4	173	404	F	0	Y		15:01	15:07	+	+	+	+	-	-	-	-	-	-	-	-	SS, MacroC
4	196	87	F	0	Y		14	11	+	+	-	-	-	-	n.a.	n.a.	-	-	-	-	OD
4	201	265	M	0	Y		13	14	+	+	-	-	-	-	n.a.	n.a.	-	-	-	-	
4	237	532	M	0	Y		13	14	+	+	-	-	-	-	n.a.	n.a.	-	-	-	-	
4	245	557	F	0	Y		10	11:05	+	+	-	-	-	-	n.a.	n.a.	-	-	-	-	NA
4	260	608	F	0	Y		34	36	+	+	+	+	-	-	-	-	-	-	-	-	
4	269	635	M	0	Y		14	15	+	+	-	-	-	-	n.a.	n.a.	-	-	-	-	
4	280	651	F	0	Y		14	15	+	+	-	-	-	-	n.a.	n.a.	-	-	-	-	
4	281	652	M	0	Y		10	12	+	+	-	-	-	-	n.a.	n.a.	-	-	-	-	
4	296	675	F	0	Y		29	29	+	+	-	-	n.a.	n.a.	-	-	-	-	-	-	
5	11	17	M	9	N	Paternal	12	15:08	+	+	-	-	-	-	-	-	-	-	-	-	Cry
5	32	170	M	3	N	Maternal	12:09	12:11	+	+	+	+	-	-	-	-	-	-	-	-	
5	40	79	F	1	N	Maternal	18	19:01	+	+	+	+	-	-	-	-	-	-	-	-	
5	62	121	F	0	N	Paternal	8:05	12	+	+	+	+	-	-	-	-	-	-	-	-	
5	92	183	F	2	N	Paternal	5	6:02	+	+	-	-	-	-	-	-	-	-	-	-	NA
5	100	212	M	0	N	Paternal	15:08	17	+	+	-	-	-	-	-	-	-	-	-	-	Thor
5	101	216	F	1	N	Maternal	7:06	8:02	+	+	+	+	-	-	-	-	-	-	-	-	LD
5	112	243	F	4	N	Paternal	10:01	10:06	+	+	-	+	-	-	-	-	-	-	-	-	Thor
5	124	269	M	2	N	Maternal	3:08	5:06	+	+	-	-	-	-	-	-	-	-	-	-	UH
5	147	323	F	2	N	Paternal	3:05	4:03	+	+	-	-	-	-	-	-	-	-	-	-	PS, Noon, IH
5	161	391	F	1	N	Paternal	10:01	10:04	+	+	-	-	-	-	-	-	-	-	-	-	
5	167	416	F	3	N	Paternal	0:07	1:01	+	+	-	-	-	-	-	-	-	-	-	-	
5	178	492	M	1	N	Paternal	5	5:03	+	+	-	-	-	-	-	-	-	-	-	-	MacroC, Noon
5	179	362	F	2	N	Paternal	8:01	9:01	+	+	+	+	-	-	-	-	-	-	-	-	IPP
5	207	335	F	0	N	Paternal	9	10	+	+	-	-	-	-	n.a.	n.a.	-	-	-	-	
5	216	638	M	2	N	Paternal	1	4	+	+	-	-	-	-	n.a.	n.a.	-	-	-	-	Chem
5	229	504	F	3	N	Paternal	9	11:09	+	+	-	-	-	-	n.a.	n.a.	-	-	-	-	SS
5	261	609	F	0	N	Maternal	32	34	+	+	+	+	-	-	n.a.	n.a.	-	-	-	-	Noon
5	262		F	0	N	Maternal	28	29	+	+	-	-	-	-	n.a.	n.a.	-	-	-	-	Noon, MacroC
5	286	658	M	1	N	Maternal	7	7:05	+	+	-	-	-	-	n.a.	n.a.	-	-	-	-	AD

^1^ The most serious clinical features that could reduce patients’ life expectancy are highlighted in bold. **Abbreviations:** AD = Attention deficit; ArC = Arachnoid cyst; AtC = Atypical CALMs; AtIN = Atypical iris nodules; Bover = Bone overgrowth; BP = Bilateral ptosis; BSL = Brain stem lesions; BvP = Behavior problems; CD = Other cardiac defects (DIA, HCM, CoA); Chem = Cherry hemangioma; CIm = Chiari I malformation; Cry = Cryptorchidism; DE = Dural ectasia; Dlip = Diffuse lipomas; DLL = Legs of different length (>1 cm); DS = Dystrophic scoliosis; Epy = Epilepsy (idiopathic); HY = Hypertension; Hyd = Hydrocephalus; ID = Intellectual disability (moderate or severe); IH = Inguinal herniation; IPP = Idiopathic precocious puberty; LD = Learning disabilities (calculation, reading, memory); Leuk = Leukemia; LGG = Low-grade glioma (other than OPG); MacroC = Macrocephaly (>95th PCTL or +3.33 plus 2 SD of difference with height); MBG = Multifocal brain gliomas; MLyn = Mild lymphoedema; MMS = Moyamoya syndrome; MPNST = Malignant peripheral nerve sheath tumor; Myo = Myopia; NA = Nevus anemicus; NBOs = Neurofibromatosis bright objects; Noon = Noonan-like facial features; OD = Oligodontia; OPG = Optic pathway glioma; PmD = Psychomotor delay; PS = Pulmonary stenosis; RMS = Rhabdomyosarcoma; SF = Spinal form; SP = Speech problem (needing therapy); SS = Short stature (<3rd PCTL; <10th PCTL general population); Thor = Thorax abnormalities (excavatum or carinatum, asymmetric); UH = Umbilical herniation; VS = Vertebral scalloping; XG = Xantogranulomas; n.a. = not available.

**Table A4:** Unique variants identified in other disease genes: *KIT* (RefSeq NM_000222.2), *LZTR1* (RefSeq NM_006767.3), *NF2* (RefSeq NM_000268.3), *PPP1CB* (RefSeq NM_206876.1), *PTEN* (RefSeq NM_000314.4), *PTPN11* (RefSeq NM_002834.3), *SOS1* (RefSeq NM_005633.3), *TSC1* (RefSeq NM_000368.4), and *TSC2* (RefSeq NM_000548.3).

Family ID ^1^	Gene	Exon	Type ^2^	Genomic	cDNA	Effect	Protein	ClinVar/HGMD/LOVD ID ^3^
240 (1)	*KIT*	14	DEL	2027del	2027del	Frame-shift	Gly676Valfs*4	New
298 (1)	*LZTR1*	4	SNV	353G>A	353G>A	Missense	Arg118His	LOVD: LZTR1_000051
232 (1)	*NF2*	5	DEL	465del	465del	Frame-shift	Ser156Valfs*18	ClinVar: 547705
238 (1)		13	SNV	1396C>T	1396C>T	Nonsense	Arg466*	ClinVar: 3295
226 (1)		2–6	DUP	(114+1_115-1)_(599+1_600-1)dup (intragenic duplication ex. 2-6)	n.a.	Intragenic duplication	?	New
263 (1)	*PPP1CB*	3	SNV	146C>G	146C>G	Missense	Pro49Arg	ClinVar: 254648
258 (2)	*PTEN*	7	INS	778_779insG	778_779insG	Frame-shift	Lys260Argfs*38	New
287 (1)	*PTPN11*	3	SNV	235C>A	235C>A	Missense	Gln79Lys	ClinVar: 44605
242 (1)		8	SNV	923A>G	923A>G	Missense	Asn308Ser	ClinVar: 13327
191 (1)		12	SNV	1403C>T	1403C>T	Missense	Thr468Met	ClinVar: 13331
204 (1)								
257 (1)								
272 (1)								
236 (1)		13	SNV	1492C>T	1492C>T	Missense	Arg498Trp	ClinVar: 40553
301 (1)			SNV	1528C>G	1528C>G	Missense	Gln510Glu	ClinVar: 40566
270 (1)	*SOS1*	4	SNV	429G>T	429G>T	Missense	Lys143Asn	New
187 (2)		10	SNV	1642A>C	1642A>C	Missense	Ser548Arg	LOVD: SOS1_000142
189 (2)	*TSC1*	13	DEL	1326_1327del	1326_1327del	Frame-shift	Gly443Ilefs*15	ClinVar: 421669
265 (1)		18	SNV	2293C>T	2293C>T	Nonsense	Gln765*	ClinVar: 48934
274 (1)		19	INS	2421dup	2421dup	Frame-shift	Ala808Cysfs*18	LOVD: TSC1_00419
305 (1)	*TSC2*	1–16	DEL	(-30+1_-29-1)_(1716+1_1717-1)del (intragenic deletion ex. 1-16)	n.a.	Intragenic deletion	?	New
267 (1)		1–22	DEL	c.(-30+1_-29-1)_(2545+1_2546-1)del (intragenic deletion ex. 1-22)	n.a.	Intragenic deletion	?	New
295 (1)		7	SNV	648+1G>T	n.a.	Splicing	?	LOVD: TSC2_03572
303 (1)		37	DEL	c.(4662+1_4663-1)_(4849+1_4850-1)del (intragenic deletion ex. 37)	n.a.	Intragenic deletion	?	LOVD: TSC2_03465
248 (1)		41	DEL	5238_5255del	5238_5255del	In-frame deletion	His1746_Arg1751del	LOVD: TSC2_00149

^1^ Number of family members presenting the variant is reported in parentheses; ^2^ Type of variant: SNV = Single-nucleotide variant, DEL = Deletion, DUP = Duplication, INS = Insertion. ^3^ ID of annotated variants in ClinVar (www.ncbi.nlm.nih.gov/clinvar), Human Genome Variation Database (HGMD; www.hgmd.cf.ac.uk) and Leiden Open Variation Database (LOVD; databases.lovd.nl/shared/genes).
