# Supplementary material for: Clinical and Genetic Findings in Children with Neurofibromatosis Type 1, Legius Syndrome, and Other Related Neurocutaneous Disorders
Source: Genes (Basel). 2019 Jul 31;10(8):580. doi: 10.3390/genes10080580 (PMC6722641; doi:10.3390/genes10080580)
Supplement: Supplementary file 1 [file genes-10-00580-s001.zip › Table S1.docx]

**Table S1** - List of primer pairs designed to amplify overlapping fragments for RNA analysis of the entire coding sequences of *NF1* and *SPRED1*

| **Exons** | **NM_000267.3 Position (bp)** | **Primer** | **Amplicon (bp)** | **Annealing temperature (°C)** |
| --- | --- | --- | --- | --- |
| 1-2 | -121F | ctgcactccacagaccctctcctt | 293 | 66 |
|  | 172R | GGCCGCTTATAACCAAAGAAAACTTGTA |  |  |
| 2-5 | 90F | ACATACCAAAGTCAGTACTGAGCACA | 509 | 65 |
|  | 598R | AAAACGACTCCTGAAGGAAACAGCATTTA |  |  |
| 5-9 | 539F | TACAGTATATCAATGTGGATTGTGCA | 456 | 65 |
|  | 994R | CTGTGTCAAACTGTGTAAAGCAAGTA |  |  |
| 9-12 | 930F | TGGAGGAAGTAGGCAGCTGACAGAAAG | 484 | 65 |
|  | 1413R | CACCGAGTCTTACATTTAAAGAAAAA |  |  |
| 12-16 | 1350F | AGTGCAAGGTTGTGGAGCACACCCAG | 485 | 65 |
|  | 1834R | TTGATCTGCAGGAATAAATTTCTTC |  |  |
| 16-18 | 1770F | GCTTAGTAGCACAGAAATTCTCAAG | 483 | 65 |
|  | 2252R | TCTGTCAGCAATATGATGTCAACAGG |  |  |
| 18-21 | 2180F | CAGTGCATAACCTCTTGCCCAACTA | 487 | 65 |
|  | 2666R | GTATCTGCGTTTCCCTCTGAAGACA |  |  |
| 21-23 | 2604F | TCCAGTCAGTGAACGTAAGGGTTCT | 530 | 65 |
|  | 3133R | GGTATTCTACCATCTTATTCCTAAATTTCATCTC |  |  |
| 23-27 | 3034F | AAAACGAAACTGTGTCAATTAGTTGAAGT | 492 | 63 |
|  | 3525R | TGTCTGGAGATCCTTGTGGTAACCT |  |  |
| 26-29 | 3461F | ATGCCAACGTAGACAGTGGTCTCAT | 495 | 65 |
|  | 3955R | AGCTAACATGTTGCCAATCAGAGGA |  |  |
| 29-31 | 3896F | AACTCCTGGATCCTTTATTACGAATTG | 294 | 64 |
|  | e30-31R | AACGCTGGCTAACCACtgattttt |  |  |
| 31-33 | e30-31F | ACTGTTTATACCAGgcaacttgcca | 327 | 64 |
|  | 4360R | CTGCATCAAAGTTGCTTTTCACAAA |  |  |
| 33-36 | 4308F | AGAAGAACATATGCGGCCTTTCAAT | 430 | 65 |
|  | 4737R | TTTGGAAGTCCCAGCTTGGTAGAAAATA |  |  |
| 35-37 | 4614F | GTCCAGCCTTAACCTTACCAGTTCAA | 500 | 65 |
|  | 5113R | GTTTCTGTTGTTCATGCTCTATGTGCTC |  |  |
| 37-39 | 5047F | AAAAGGCTTGTTTTCATAGACTGTCCTG | 519 | 65 |
|  | 5565R | AAGATTATAGGCAGCTGACCGTAAACTC |  |  |
| 38-40 | 5464F | ACCAAGATTCGGCCAAAAGATGTCC | 480 | 68 |
|  | 5943R | CTGCCCAAGGCTTCCCCATATTTT |  |  |
| 39-42 | 5862F | TGACAAGCTGATAACAATGACCATCA | 519 | 65 |
|  | 6380R | ACTTGCTTGGTCTCTTCACTAAAATGAAG |  |  |
| 41-44 | 6295F | GCTTCCACACATGGACTGGTCATTA | 480 | 65 |
|  | 6774R | TAAGCAACTCTCAAGTGCCTTGCTAAGA |  |  |
| 44-48 | 6680F | CTCTTGTTGTCTTTGGGTGTATTAGCAA | 485 | 65 |
|  | 7164R | TGTTCTTGCAACAATAGCAGGTGAAG |  |  |
| 47-51 | 7099F | TTTGCATTGGTTGGACACCTTTTA | 500 | 64 |
|  | 7598R | GGAGCCTTTGTGTCTGATATCAAGTG |  |  |
| 50-54 | 7523F | CTTCTCAGGCCAACACTAAGAAGTTGCT | 497 | 65 |
|  | 8019R | GGATTCTTCATGGTACACCACACTCTG |  |  |
| 54-57 | 7956F | AGATCCAAATTTGTTAAATCCAATCC | 549 | 62 |
|  | 8550R | tgaagagcccatgttaagttgatttt |  |  |
| **Exons** | **NM_152594.3 Position (bp)** | **Primer** | **Amplicon (bp)** | **Annealing temperature (°C)** |
| 1-6 | -155F | taccgttctgggtgaggcatcc | 850 | 65 |
|  | 695R | TTCAAAGGGACCCTATTTTGGGACTT |  |  |
| 5-7 | 573F | AGCCAATCAGATAACATTTGGTCAGC | 839 | 65 |
|  | 1411R | ccaaaagcttccacaaatccaagtta |  |  |
