# Supplementary material for: Clinical and Genetic Findings in Children with Neurofibromatosis Type 1, Legius Syndrome, and Other Related Neurocutaneous Disorders
Source: Genes (Basel). 2019 Jul 31;10(8):580. doi: 10.3390/genes10080580 (PMC6722641; doi:10.3390/genes10080580)
Supplement: Supplementary file 1 [file genes-10-00580-s001.zip › Table S2.docx]

**Table S2 -** List of genomic primer pairs designed to amplify exons and intronic flanking regions of *NF1* and *SPRED1*

| ***NF1* (Reference genomic sequence NG_009018.1)** | | | | | | | | | | |
| --- | --- | --- | --- | --- | --- | --- | --- | --- | --- | --- |
| **Exon** |  | **Primer** | **Amplicon**  **(bp)** | **Annealing temperature (°C)** |  | **Exon** |  | **Primer** | **Amplicon (bp)** | **Annealing temperature (°C)** |
| 1(1) | F | ctagtggggagagcgaccaagag | 409 | 65 |  | 30(23.1) | F | gttttaaacaaaagtgttaggatttt | 240 | 56 |
|  | R | ctcctacctcccctcacctactctgt |  |  |  |  | R | aaagcattcttcataatttaactttag |  |  |
| 2(2) | F | ggcaagtaagttatttatggtcgttt | 249 | 60 |  | 31(23.2) | F | ctgtatgtagtcggtgctgtgact | 155 | 60 |
|  | R | aaattccccaaaacacagtaacc |  |  |  |  | R | tgctttgaaagaaacagctaataaaa |  |  |
| 3(3) | F | tgattggtagcagaaagtgaaactaa | 180 | 60 |  | 32(24) | F | caaaccttatactcaattctcaactcc | 238 | 60 |
|  | R | aaattcacaaagcctgcctactta |  |  |  |  | R | ggaaggggaatttaagatagctagat |  |  |
| 4(4a) | F | tttcataatagaaaatgtttacaggta | 297 | 56 |  | 33(25) | F | aatctgattatttataaccctgtttt | 199 | 56 |
|  | R | catattacttcagtagtcccatgtg |  |  |  |  | R | ggatttatgtgaaaccgaaaata |  |  |
| 5(4b) | F | agataccacacctgtcccctaatact | 226 | 60 |  | 34(26) | F | tctggtgttgaaaattctaatgactt | 237 | 59 |
|  | R | tgatactagtttttgacccagtgatt |  |  |  |  | R | tctaaatttaaacggagagtgttcac |  |  |
| 6(4c) | F | tttgctctgagttgtatttgtgttaa | 199 | 59 |  | 35(27a) | F | aaatcagctgacagtaaaaggaaaag | 289 | 60 |
|  | R | atgctaacaacagcaaattttacatc |  |  |  |  | R | aagaaagcaatcaactatttcaaggt |  |  |
| 7(5) | F | aaaatcactgtaaagacatgtggttc | 195 | 60 |  | 36(27b) | F | actcagtagacaacataaagcctcat | 197 | 58 |
|  | R | cttggaaaacgatgataggtttttat |  |  |  |  | R | ttctgttaagagacccaaaacatagt |  |  |
| 8(6) | F | ccagggattttgttcctatctaataa | 254 | 60 |  | 37(28) | F | gcatctgtatatttattttaaacactgct | 530 | 59 |
|  | R | tgcttcacatctagatataatggaaat |  |  |  |  | R | aatgaatcgtttacaaaacacagact |  |  |
| 9(7) | F | aagaaacttcatatattatcttatcgctat | 294 | 57 |  | 38(29) | F | agagtttaattcttctccacttcac | 423 | 57 |
|  | R | ttgacataatacttatgctagaaaattcat |  |  |  |  | R | cccaaatcaaactgaagagaat |  |  |
| 10(8) | F | ggtatttaaaggcttttgttttctgt | 223 | 59 |  | 39(30) | F | ttttggaactataaggaaaaatacgtt | 326 | 59 |
|  | R | tttgtttataaaggataacagcatcag |  |  |  |  | R | gaaagggttttctttgaattctctta |  |  |
| 11(9) | F | tttttctcatagaaataatctgctttt | 195 | 57 |  | 40(31) | F | taattgttgatgtgattttcattgac | 297 | 59 |
|  | R | ttcttaaaaagaaatacgcaaagaa |  |  |  |  | R | tgtgcacaaaggagatactagataaa |  |  |
| 12(10a) | F | ttttgtactttttcttcctattggtc | 212 | 59 |  | 41(32) | F | aaattggtagagtgattaaaaacatg | 247 | 58 |
|  | R | agaaaggaggtgagattcaaaattc |  |  |  |  | R | aacctcctgatgataaaacagatatg |  |  |
| 13(10b) | F | cctgagtcttatgtctgataccatgt | 240 | 59 |  | 42(33) | F | gtgctaaaactttgagtcccatgt | 393 | 60 |
|  | R | ttcagctaaacccaattaacttagtg |  |  |  |  | R | agtaaaatggagaaaggaactggtaa |  |  |
| 14(10c) | F | tcctccttctaatctctctcgattta | 219 | 60 |  | 43(34) | F | tcaaaatgaaacatggaactttagaa | 398 | 60 |
|  | R | aatctttccttcaagaacatggaat |  |  |  |  | R | gcattaagtacaaaatagcacaataaacc |  |  |
| 15(11) | F | ttaccaaaaatgtttgagtgagtctt | 212 | 58 |  | 44(35) | F | tgacatcataataaacattatttaaacag | 200 | 57 |
|  | R | cagagttaatatttcaagacaaaaataca |  |  |  |  | R | atagctgtattttcaacaatatgca |  |  |
| 16(12a) | F | ggttattgatgatgctagtaacaatga | 219 | 60 |  | 45(36) | F | caaaggtttttataagttctgtggatc | 196 | 60 |
|  | R | cattaccattccaaatattcttccat |  |  |  |  | R | agaaccataaatatttgggagaagtg |  |  |
| 17(12b) | F | ttgtcagtgcttcagtaaagcttatt | 257 | 60 |  | 46(37) | F | ttccgagattcagtttaggagttaat | 219 | 60 |
|  | R | attcagaaaacaaacagagcacataa |  |  |  |  | R | ctgatacccaaaatgaatgcact |  |  |
| 18(13) | F | tacccaagttgcaaatatatgtcttc | 338 | 60 |  | 47(38) | F | tgattcatcttactagcctcaaacat | 250 | 60 |
|  | R | gtgctttgaggcagactgagtaa |  |  |  |  | R | agcaacaagaaaagatggaagagtac |  |  |
| 19(14) | F | atttgctctgctcttcctactccttt | 248 | 60 |  | 48(39) | F | aagaaagctactgtgtgaacctcat | 248 | 60 |
|  | R | tatgtaacaagaggcttccaaattga |  |  |  |  | R | cacttattcaaattacttctggtttcttaa |  |  |
| 20(15) | F | gctctagactaagttgctttcaagtg | 169 | 59 |  | 49(40) | F | agttgttagtcagggaagaagacct | 295 | 60 |
|  | R | cagatcagttaacagacaaaagtcaa |  |  |  |  | R | gtatttgtactttttgggtgatcctt |  |  |
| 21(16) | F | ttgtcaagtctcaactaattaaggtt | 540 | 56 |  | 50(41) | F | tcacacttgtgatttgttaaattttt | 243 | 59 |
|  | R | tattcatagagaaaggtgaaaaataa |  |  |  |  | R | tggaaaattgaaacttgattatgatt |  |  |
| 22(17) | F | agatcagtcagtttcatctctctagg | 297 | 57 |  | 51(42) | F | ggttgtatttgtcaccatattaattga | 295 | 59 |
|  | R | actaccagtatcagtgtgtaagaggt |  |  |  |  | R | gaaatttcaaataattttccaagagtaa |  |  |
| 23(18) | F | atgccttctcttttgtctatatctga | 219 | 60 |  | 52(43) | F | ttcccatttatagacactgtagttaatga | 240 | 60 |
|  | R | agatttacaagaccctacattgctct |  |  |  |  | R | actttcatgtactctcccaccttatt |  |  |
| 24(19a) | F | acgtgactaaaggtgtgtgtgtg | 196 | 59 |  | 53(44) | F | tatccaggtgtttgatcacgttaat | 238 | 60 |
|  | R | cagtaaaacccactaatacttgaagg |  |  |  |  | R | gcctcctaaaagtagactggaataaa |  |  |
| 25(19b) | F | cttgaaagattcatggtctctaaat | 214 | 56 |  | 54(45) | F | ataacaattcagccacaaagtaaaaa | 195 | 60 |
|  | R | tttatttgctttttgctttatgt |  |  |  |  | R | cacgaaggtgaattaaaatcaaaag |  |  |
| 26(20) | F | gccttcactatgtaaaggtcagtctt | 276 | 60 |  | 55(46) | F | cctctaaaatgttcctctgttgactt | 231 | 60 |
|  | R | tacatgccagttctctaggttttgta |  |  |  |  | R | catgttagcaagttcatcaaccat |  |  |
| 27(21) | F | taagagaagcaaaaattacttcagca | 341 | 60 |  | 56(47) | F | tgttacaattaaaagataccttgcttgt | 168 | 60 |
|  | R | tatattctgaaggatttgctatgtgc |  |  |  |  | R | aggcatactaatttgaacagaaacct |  |  |
| 28(22) | F | attgtttgcactaacctgattttgt | 254 | 60 |  | 57(48) | F | cttcagatggggatttacttaaaaa | 315 | 59 |
|  | R | aatcattacttgacatacctcagcac |  |  |  |  | R | tgttgctcaaagtcatataaagatca |  |  |
| 29(23) | F | aggtataataaactcctattcgtgca | 215 | 59 |  | 58(49) | F | aggaaaagaagaagtaactggctgt | 296 | 60 |
|  | R | catgcagtgttagtaaaacaaacaaa |  |  |  |  | R | tataaaacaggaagtgcagcattaca |  |  |
| ***SPRED1* (Reference genomic sequence NG_008980.1)** | | | | | | | | | | |
| **Exon** |  | **Primer** | **Amplicon (bp)** | **Annealing temperature (°C)** |  | **Exon** |  | **Primer** | **Amplicon (bp)** | **Annealing temperature (°C)** |
| 1 | F | cggagctctgcttcctcctc | 604 | 60 |  | 5 | F | ttgtggtggtggtggttttt | 352 | 60 |
|  | R | ctccagaagatgcaccgaac |  |  |  |  | R | ttttgtttgtggggtttttg |  |  |
| 2 | F | caaacaagactgatggcttgg | 376 | 60 |  | 6 | F | atgaggttttggaacatacactg | 316 | 59 |
|  | R | cacagaaacagctccagaaaca |  |  |  |  | R | ggcagcaaatttatacaatgaaaa |  |  |
| 3 | F | agcgttgtatcacctcagtttg | 398 | 60 |  | 7a | F | tgctttcctcatagtccacca | 552 | 60 |
|  | R | tgaggtttcaaagcctggtc |  |  |  |  | R | ggagcatcctgacattttcc |  |  |
| 4 | F | ttaattgccaggcagtccag | 335 | 60 |  | 7b | F | agacgcagccttcctcatta | 452 | 59 |
|  | R | ggatgctcaacctgtattgg |  |  |  |  | R | agctaattcctggaaacaaaga |  |  |
