# Supplementary material for: Clinical and Genetic Findings in Children with Neurofibromatosis Type 1, Legius Syndrome, and Other Related Neurocutaneous Disorders
Source: Genes (Basel). 2019 Jul 31;10(8):580. doi: 10.3390/genes10080580 (PMC6722641; doi:10.3390/genes10080580)
Supplement: Supplementary file 1 [file genes-10-00580-s001.zip › Table S3.docx]

**Table S3 -** List of genes included in the customized target NGS panel

| ***A2ML1*** | *ITPR1* | *MCM2* | *PDGFRB* | ***PTEN*** | ***RIT1*** | *SNAI2* |
| --- | --- | --- | --- | --- | --- | --- |
| ***ANTXR2*** | ***KAT6B*** | ***MLH1*** | *PMS1* | ***PTPN11*** | *RNF135* | *SOCS6* |
| *BLM* | *KHDRBS1* | *MLH3* | ***PMS2*** | *RAD17* | *RPA1* | ***SOS1*** |
| ***BRAF*** | ***KIT*** | *MRVI1* | ***POGLUT1*** | *RAD52* | *RPA2* | ***SOS2*** |
| ***CBL*** | *KITLG* | ***MSH2*** | *POLA1* | ***RAF1*** | *RPA3* | ***SPRED1*** |
| *CD2AP* | ***KRAS*** | *MSH3* | *POLD1* | *RALGDS* | *RPS6KA3* | *SPRED2* |
| *CHTF18* | *KSR1* | *MSH4* | *POLD2* | *RASA1* | ***RRAS*** | *SPRY1* |
| ***EPCAM*** | *LIG1* | ***MSH6*** | *POLD3* | ***RASA2*** | *SH3KBP1* | *SPRY2* |
| *EXO1* | ***LZTR1*** | ***NF1*** | *POLD4* | *RFC1* | ***SHOC2*** | *SPRY3* |
| *FEN1* | ***MAP2K1*** | ***NF2*** | *POLE* | *RFC2* | *SMARCA4* | *SUZ12* |
| *GAB1* | ***MAP2K2*** | ***NOTCH3*** | *PPP1CA* | *RFC3* | ***SMARCB1*** | ***TSC1*** |
| *GRB2* | *MAP3K8* | ***NRAS*** | ***PPP1CB*** | *RFC4* | *SMARCC1* | ***TSC2*** |
| *GUCY1A3* | *MAPK1* | *PAK3* | *PPP1CC* | *RFC5* | *SMARCE1* | *YWHAB* |
| ***HRAS*** | *MAPK3* | *PCNA* | *PRKG1* | *RHEB* | *SNAI1* |  |

Note: Disease-causing genes are highlighted in bold.
