# Supplementary material for: Clinical and Genetic Findings in Children with Neurofibromatosis Type 1, Legius Syndrome, and Other Related Neurocutaneous Disorders
Source: Genes (Basel). 2019 Jul 31;10(8):580. doi: 10.3390/genes10080580 (PMC6722641; doi:10.3390/genes10080580)
Supplement: Supplementary file 1 [file genes-10-00580-s001.zip › Table S4.docx]

**Table S4 -** *In silico* prediction of deleterious effects and segregation analysis for unreported missense variants

| **Gene** | **Mutation** | **Protein** | **SIFT** | **PolyPhen-2** | **Mutation T@ster** | **Segregation** |
| --- | --- | --- | --- | --- | --- | --- |
| *NF1* | 818T>C | Leu273Pro | Affect protein function | Probably damaging (score 1.000) | Disease causing (score: 0.977) | *de novo* |
|  | 1144T>C | Ser382Pro | Affect protein function | Probably damaging (score 0,999) | Disease causing (score: 0.999) | *de novo* |
|  | 1487T>G | Met496Arg | Affect protein function | Possibly damaging (score 0.523) | Disease causing (score: 0.999) | familiar |
|  | 3104T>A | Met1035Lys | Affect protein function | Probably damaging (score 0.962) | Disease causing (score: 0.997) | *de novo* |
|  | 3106A>G | Lys1036Glu | Affect protein function | Possibly damaging (score 0.893) | Disease causing (score: 0.999) | *de novo* |
|  | 3592G>A | Glu1198Lys | Affect protein function | Possibly damaging (score 0.605) | Disease causing (score: 0.999) | familiar |
|  | 5437T>C | Ser1813Pro | Affect protein function | Probably damaging (score 0.968) | Disease causing (score: 0.999) | *de novo* |
|  | 5676G>T | Lys1892Asn | Affect protein function | Probably damaging (score 0.989) | Disease causing (score: 0.999) | *de novo* |
| *SOS1* | 429G>T | Lys143Asn | Affect protein function | Probably damaging (score 1.000) | Disease causing (score: 0.999) | *de novo* |
