# Supplementary material for: Clinical and Genetic Findings in Children with Neurofibromatosis Type 1, Legius Syndrome, and Other Related Neurocutaneous Disorders
Source: Genes (Basel). 2019 Jul 31;10(8):580. doi: 10.3390/genes10080580 (PMC6722641; doi:10.3390/genes10080580)
Supplement: Supplementary file 1 [file genes-10-00580-s001.zip › Table S5.docx]

**Table S5 -** *In silico* prediction of splice score for deep intronic mutations in *NF1* (www.fruitfly.org/seq_tools/splice.html)

| **Deep intronic mutation** | **Hg19 coordinates on Chr 17 of retained intronic regions and splice score** | | | | | **ClinVar/HGMD/LOVD ID^1^** |
| --- | --- | --- | --- | --- | --- | --- |
| c.289-2956C>T |  | 29487129 |  | 29487246 |  | New |
|  | atatttttcttttgagacag | **agtctcactctgcggcacag** | … 78 bp … | **ccaagtagctgggattacag** | gcatgtaccaccatgctcag |  |
|  | *Splice score (0.0)* |  |  |  | **t** *Splice score (1.0)* |  |
| c.1260+1604A>G |  | 29530108 |  | 29530149 |  | LOVD: NF1_000035 |
|  | tgaaatatttttgtctacaa | **ctgactacatagagcactt** | … 2 bp … | **agcatggacttggcactgct** | gtaagtggctgagctgctcc |  |
|  | *Splice score (0.0)* **G** |  |  |  | *Splice score (0.96)* |  |
| c.1393-1554C>G |  | 29539842 |  | 29539913 |  | New |
|  | tttttttatatgactagaag | **tgaagatttgtttacaccag** | … 32 bp … | **acatcacgatggctatgata** | ctaagtgataggaatttttc |  |
|  | *Splice score (0.0)* |  |  |  | **g** *Splice score (0.97)* |  |
| c.1527+1165T>A |  |  |  |  |  | New |
|  | ctatattgtgtctttcatag | **gatgacatgtttaacctttg** | … 19 bp … | **ggagagcagcatcaagcaag** | gtttcttatcgttttgctca |  |
|  | *Splice score (0.61)* |  |  |  | **a** *Splice score (0.52)* |  |

Notes: (1) ID of annotated variants in ClinVar (www.ncbi.nlm.nih.gov/clinvar), Human Genome Variation Database (HGMD; www.hgmd.cf.ac.uk) and Leiden Open Variation Database (LOVD; databases.lovd.nl/shared/genes/NF1).
