# Supplementary material for: Clinical and Genetic Findings in Children with Neurofibromatosis Type 1, Legius Syndrome, and Other Related Neurocutaneous Disorders
Source: Genes (Basel). 2019 Jul 31;10(8):580. doi: 10.3390/genes10080580 (PMC6722641; doi:10.3390/genes10080580)
Supplement: Supplementary file 1 [file genes-10-00580-s001.zip › Figure S1.docx]

**Figure S1 –** Distribution of identified variants in exons of *NF1*

Causative variants in unrelated NF1 patients were annotated according to exonic organization of *NF1*. Variants were distributed in almost all exons and particularly enriched in exons 12(10a), 13(10b), 38(29) and 46(37). A total of 21 were recurrent variants, being present in at least two unrelated patients.
